# Time-resolved transcriptome and proteome landscape of human regulatory T cell (Treg) differentiation reveals novel regulators of FOXP3

**DOI:** 10.1186/s12915-018-0518-3

**Published:** 2018-05-07

**Authors:** Angelika Schmidt, Francesco Marabita, Narsis A. Kiani, Catharina C. Gross, Henrik J. Johansson, Szabolcs Éliás, Sini Rautio, Matilda Eriksson, Sunjay Jude Fernandes, Gilad Silberberg, Ubaid Ullah, Urvashi Bhatia, Harri Lähdesmäki, Janne Lehtiö, David Gomez-Cabrero, Heinz Wiendl, Riitta Lahesmaa, Jesper Tegnér

**Affiliations:** 1 Unit of Computational Medicine, Center for Molecular Medicine, Department of Medicine Solna, Karolinska Institutet, Karolinska University Hospital and Science for Life Laboratory, 17176 Stockholm, Sweden; 2 Department of Signaling and Gene Expression, La Jolla Institute for Allergy and Immunology, La Jolla, CA 92037 USA; 3 Neuroimmunology Unit, Center for Molecular Medicine, Department of Clinical Neuroscience, Karolinska Institutet & Karolinska University Hospital, 17176 Stockholm, Sweden; 4 Department of Neurology, University Hospital Münster and University of Münster, 48149 Münster, Germany; 5 Department Oncology-Pathology, Cancer Proteomics Mass Spectrometry, Science for Life Laboratory, Karolinska Institutet, 17176 Stockholm, Sweden; 6 Department of Computer Science, Aalto University, FI-00076 Aalto, Finland; 7 Turku Centre for Biotechnology, University of Turku and Åbo Akademi University, FI-20521 Turku, Finland; 8 Mucosal and Salivary Biology Division, King’s College London Dental Institute, London, SE1 9RT UK; 9 Biological and Environmental Sciences and Engineering Division, Computer, Electrical and Mathematical Sciences and Engineering Division, King Abdullah University of Science and Technology (KAUST), Thuwal, 23955-6900 Kingdom of Saudi Arabia

**Keywords:** Regulatory T cells, Treg, iTreg, FOXP3, T cell differentiation, RNA sequencing (RNA-Seq), Proteomics, Data integration, TGF-β

## Abstract

**Background:**

Regulatory T cells (Tregs) expressing the transcription factor FOXP3 are crucial mediators of self-tolerance, preventing autoimmune diseases but possibly hampering tumor rejection. Clinical manipulation of Tregs is of great interest, and first-in-man trials of Treg transfer have achieved promising outcomes. Yet, the mechanisms governing induced Treg (iTreg) differentiation and the regulation of FOXP3 are incompletely understood.

**Results:**

To gain a comprehensive and unbiased molecular understanding of FOXP3 induction, we performed time-series RNA sequencing (RNA-Seq) and proteomics profiling on the same samples during human iTreg differentiation. To enable the broad analysis of universal FOXP3-inducing pathways, we used five differentiation protocols in parallel. Integrative analysis of the transcriptome and proteome confirmed involvement of specific molecular processes, as well as overlap of a novel iTreg subnetwork with known Treg regulators and autoimmunity-associated genes. Importantly, we propose 37 novel molecules putatively involved in iTreg differentiation. Their relevance was validated by a targeted shRNA screen confirming a functional role in FOXP3 induction, discriminant analyses classifying iTregs accordingly, and comparable expression in an independent novel iTreg RNA-Seq dataset.

**Conclusion:**

The data generated by this novel approach facilitates understanding of the molecular mechanisms underlying iTreg generation as well as of the concomitant changes in the transcriptome and proteome. Our results provide a reference map exploitable for future discovery of markers and drug candidates governing control of Tregs, which has important implications for the treatment of cancer, autoimmune, and inflammatory diseases.

**Electronic supplementary material:**

The online version of this article (10.1186/s12915-018-0518-3) contains supplementary material, which is available to authorized users.

## Background

Immunological tolerance to self and innocuous foreign antigens is maintained by a fine-tuned balance of several immune cells, with an indispensable non-redundant role for both thymic regulatory T cells (tTregs) and peripherally induced Tregs (pTregs) [1–3]. Naturally-occurring Tregs (nTregs) comprise tTregs and pTregs, both of which suppress other immune cells and express the transcription factor (TF) Forkhead Box P3 (FOXP3). FOXP3 is necessary to generate the full Treg signature and functionality, and mutations in FOXP3 lead to severe lethal autoimmune disease in scurfy mice and men [4]. Significant progress has been made in elucidating the architecture of the regulatory elements in the FOXP3 gene locus, which responds primarily to TCR, CD28, IL-2, and TGF-β signaling pathways [5]. However, most known regulators of FOXP3 are not specific to Tregs but are, rather, factors of general importance in T cells and other immune cells. FOXP3 regulation is incompletely understood particularly in the human system despite accumulating evidence for differing FOXP3 regulation in mice versus human such as activation-induced low-level FOXP3 expression in human but not murine conventional T cells [4, 6, 7] and expression of different FOXP3 isoforms in human Tregs [8–11].

Therapeutic manipulation of endogenous Tregs and adoptive transfer of Tregs are promising current strategies for the treatment of human autoimmune and inflammatory diseases such as type 1 diabetes (T1D) and graft-versus-host disease (GvHD), respectively [12, 13]. Tregs can also be recruited to or be induced at tumor sites, where suppression of anti-tumor immune responses can be detrimental; therefore, depletion of Tregs is currently in trials for cancer treatment [14, 15]. Thus, the further understanding of Treg induction and regulation of FOXP3 expression is highly relevant to expand the therapeutic opportunities for autoimmune and inflammatory diseases as well as cancer. Due to their involvement in different physiological functions, specific targeting of pTregs or tTregs may be warranted depending on the type of disease. In vivo, pTreg generation was shown to occur in the intestinal system, where chronic low-dose antigen stimulation under tolerogenic conditions favors Treg induction [1]. Gut-associated dendritic cells as well as macrophages in the lung secrete TGF-β and the vitamin A metabolite all-trans retinoic acid (ATRA), which promote Treg induction [1, 16, 17]. Further, commensal microbiota-derived short-chain fatty acids, particularly butyrate, were demonstrated to favor colonic Treg induction [18, 19]. Specific depletion of pTregs in C57BL/6 mice leads to spontaneous development of allergic-type pathologies at mucosal sites in the gastro-intestinal tract and lung [20] as well as to defects in maternal-fetal tolerance in the placenta [21], but not to systemic autoimmune disease. This is concordant with the concept that tTregs are primarily needed to mediate self-tolerance, while pTregs are specifically induced at environmental interfaces to induce tolerance to foreign antigens. Nevertheless, a potential role for pTregs in mediating self-tolerance has also been suggested [22, 23].

Interestingly, Tregs induced from naïve T cells in vitro are able to take over nTreg functions, as they are able to rescue scurfy mice [24]. Such in vitro induced Tregs (iTregs) can be generated by protocols that mimic the in vivo situation, usually containing IL-2 and TGF-β [1, 16]. Depending on the induction protocol used, iTregs can highly express FOXP3 beyond activation-induced levels, although the epigenetic profile and, consequently, the stability of iTregs differs from that of nTregs [25, 26]. Further, the suppressive functionality of iTregs is controversial, with results dependent on the protocol and controls used [27, 28]. However, to understand molecular events occurring prior to and during FOXP3 induction in human T cells, in vitro culture systems are required.

Thus, to further understand the regulation and induction of FOXP3 we performed deep molecular profiling over time during Treg induction, starting from naïve CD4+ T cells. Since there is no ‘gold standard’ protocol for human Treg induction, we aimed to find general FOXP3 regulators independent of a specific procedure. Therefore, we used control (‘Mock’) stimulated cells along with four different recently established Treg-inducing protocols in parallel [28]. These protocols, utilizing the cytokines IL-2 and TGF-β in combination with ATRA, ATRA + rapamycin (Rapa), or butyrate, led to robust and reproducible induction of FOXP3 and other Treg signature molecules [28, 29]. Notably, the mTOR inhibitor Rapa proved efficient in enhancing iTreg generation and nTreg expansion [12, 30, 31], and we previously demonstrated that the combination of TGF-β + ATRA + Rapa induced iTregs with superior suppressive activity [28].

We present here an extensive time-series molecular profiling of human iTreg differentiation, providing a resource of RNA sequencing (RNA-Seq) transcriptomics and proteomics data covering multiple Treg-inducing protocols alongside with control cells that were activated without Treg-inducing factors. We also provide unstimulated naïve CD4+ T cells and nTregs from the same donors as negative and positive controls, respectively. We explored the transcriptome and proteome of the very same samples, enabling true integrative analysis of both data types and making deep quantitative mass spectrometry-based proteomics data of iTregs available to the scientific community. In addition, we provide a completely independent additional RNA-Seq dataset for TGF-β + ATRA iTregs generated in another laboratory under different culture conditions and with an extended time-series. We present evidence for iTreg-specific global cell polarization patterns in agreement with available relevant T cell signatures, and we find that functional categories underlying differentiation include a super-cluster of general molecular pathways related to cellular proliferation and metabolism, in parallel to a super-cluster linked to T cell polarization. Importantly, integration of our data revealed enrichment of a newly defined iTreg subnetwork for immune disease-associated genes and a central position in the iTreg subnetwork for many known crucial (n)Treg regulators, alongside with novel candidate molecules whose functionality in FOXP3 induction was confirmed by a targeted shRNA validation screen. Our rich data harbor the potential to reveal novel markers for distinction of Tregs from activated T cells or pTregs from tTregs, the latter being currently problematic in the human system [1, 32]. Indeed, our computational approach confirmed that novel iTreg molecules discovered in this study can outperform known Treg regulators in classifying iTregs versus activated T cells. In summary, we present a large resource of dynamic Treg transcriptome and proteome data, including several novel regulators of FOXP3, which can be explored as potential markers and drug targets in the future.

## Results

### Human in vitro generated iTregs show robust expression of Treg signature molecules such as FOXP3 and Eos

**Fig. 1:**
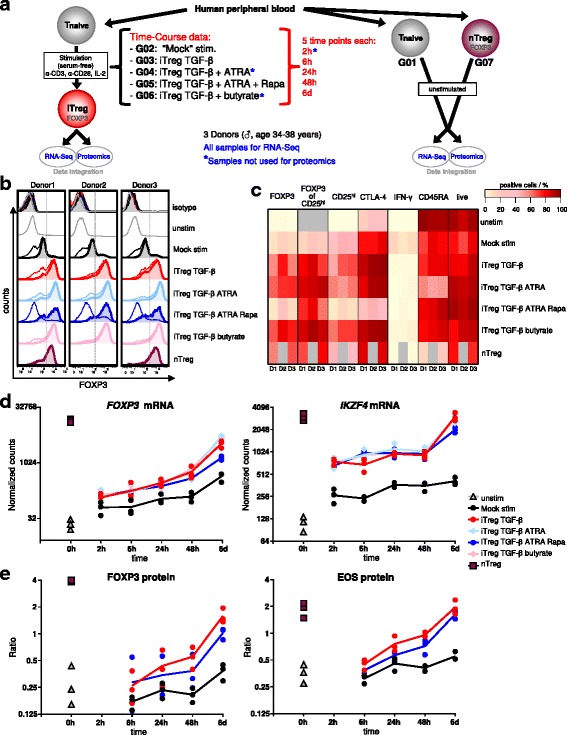
Treg signature molecules confirm the quality of iTreg and control cells used for molecular profiling. **a** Human naïve CD4+ T cells were stimulated (‘stim.’) with anti-CD3/-CD28 antibodies and IL-2 for up to 6 days in serum-free medium. For iTreg generation, TGF-β1, rapamycin (Rapa), all-trans retinoic acid (ATRA), or butyrate were added. At indicated time points (h: hours, d: days), RNA and protein were extracted for RNA-Seq and proteomics. Unstimulated (‘unstim.’) nTregs (ex vivo CD25^high^ cells) were used as a positive control, unstimulated naïve CD4+ T cells were used as the ‘time zero’ control, both harvested on the day of isolation. G01–G07: Treatment group abbreviations. **b**, **c** Cultures as in (**a**), except unstimulated cells cultured in medium (+ IL-2) for 6 days. On day 6, an aliquot was re-stimulated for 4 h with phorbol 12-myristate 13-acetate/ionomycin plus Brefeldin A, stained for the given surface and intracellular markers and analyzed by flow cytometry. **b** Histograms for FOXP3 are shown for individual molecular profiling donors. Pre-gating: live CD4 + CD25++ cells (filled histograms) or live CD4+ cells (open histograms). ‘isotype’: anti-FOXP3 antibody isotype staining for each sample colored as beneath. Grey dashed lines: gate to determine FOXP3+ cell fraction; corresponding values are displayed in (**c**). **c** Expression of flow cytometry markers, gated on viable CD4+ cells (except for last three columns depicting live cell fraction, and columns 4–6, which are gated on live CD4 + CD25++ cells). The percentage of positive cells for the given marker is indicated by the color scale, columns show individual donors (D1, D2, D3). Grey indicates samples not measured or not applicable markers. FOXP3 and *IKZF4* (Eos) expression from RNA-Seq (**d**) and proteomics (**e**) data, respectively. Dots: individual donors (mean per donor for proteomics samples with technical replicates), lines: mean of *n* = 3 donors. Statistical analysis, see Methods and Additional file 3: Table S2

To gain a better understanding of human FOXP3 induction, we induced iTregs from naïve CD4+ T cells allowing for the detection of iTreg signatures at the differentiated state but also of events preceding FOXP3 expression, which in the human system is only possible with in vitro assays. We performed a time-course molecular profiling during human iTreg induction, capturing molecular events occurring during differentiation of FOXP3+ cells along with control cells. It is well-known that TGF-β and IL-2 contribute to the induction of FOXP3 [1, 5]; however, there is no single standard protocol for iTreg generation and the literature concerning iTreg phenotype and function is controversial even for seemingly similar protocols [27, 28]. To identify robust and generic molecular events driving FOXP3 induction, we interrogated iTregs induced by four different protocols in parallel. This approach enabled stringent filtering for shared events occurring in all FOXP3-expressing iTreg populations independently of the specific protocol. The Treg-inducing protocols all included anti-CD3/-CD28 activation together with IL-2 and TGF-β, either alone or in combination with ATRA, ATRA + Rapa, or butyrate (Fig. 1a). Importantly, cells were cultured in serum-free medium to exclude traces of serum-derived TGF-β or other undefined factors, and as a control we used Mock-stimulated cells treated only with anti-CD3/-CD28 activation and IL-2. We isolated naïve CD4+ T cells and CD25^high^ cells (here called ‘nTreg’) in parallel from the same three healthy male donors (for cell purities, see Additional file 1: Figure S1a). We isolated RNA and protein from the same cells, which were derived from iTregs and Mock-stimulated cells at five different time points of differentiation (2, 6, 24, and 48 h, and 6d) and from unstimulated naïve CD4+ T cells (‘Tnaive’) and nTregs (for experimental setup, see Fig. 1a). For quality control, an aliquot of the cells used for molecular profiling was phenotyped by flow cytometry on days 4 and 6 of culture. All iTregs displayed expression of FOXP3 that was enriched in CD25-expressing cells, unlike Mock-stimulated cells (Fig. 1b, c and Additional file 1: Figure S1b, c). In addition, we determined that CD45RA was downregulated more strongly in iTreg TGF-β + ATRA as compared to the other stimulated cultures (Fig. 1c and Additional file 1: Figure S1c). Furthermore, the fraction of IFN-γ-producing cells was reduced in iTregs compared to Mock-stimulated cells (Fig. 1c). As we described previously [28], CD25 was upregulated upon stimulation resulting in a high fraction of positive cells in Mock-stimulated cells and iTregs, except in iTreg populations induced in the presence of Rapa, which displayed a generally less activated phenotype (Fig. 1b, c and Additional file 1: Figure S1c). Accordingly, after pre-gating on CD25+ cells, iTregs induced in the presence of Rapa were more similar to iTregs induced with other protocols not only regarding expression of FOXP3 but also of CTLA-4 and CD45RA (Additional file 1: Figure S1c). Notably, despite lower fractions of FOXP3+ cells in iTregs generated with TGF-β + ATRA + Rapa, these iTregs had superior suppressive function in vitro according to our previously published results [28].

High-quality RNA extracted from different time points of T cell cultures was subjected to RNA-Seq, which confirmed enhanced expression of *FOXP3* in all iTregs compared to Mock-stimulated cells at all time points (Fig. 1d). *IKZF4* encoding for Eos, another gene important for Treg function [33], was also early and stably upregulated in all iTreg populations, reaching levels similar to nTregs (Fig. 1d). *FOXP3* and *IKZF4* expression results from RNA-Seq were confirmed by qRT-PCR from the same as well as additional donors (Additional file 1: Figure S1d) [28]. From a subset of the samples, we performed quantitative mass spectrometry-based proteomics using high resolution isoelectric focusing (HiRIEF) nanoLCMS [34]. The proteomics data confirmed high expression of FOXP3 and Eos protein in iTregs induced with TGF-β or TGF-β + ATRA + Rapa (Fig. 1e). Although FOXP3 expression in both RNA-Seq and proteomics data increased over time in iTregs, reflecting the increased fraction of FOXP3+ cells in the population as differentiation proceeds, the amounts remained below that in nTreg populations. Notably, on the per-cell level, when gating on activated (CD25+) cells, FOXP3 protein levels in iTregs were similar to nTregs, while Mock-stimulated cells did not display such FOXP3 expression even in CD25++ cells (Fig. 1b, c), emphasizing the importance of considering the fraction of CD25+ cells as well as the kinetics of gene expression over time in comparison to Mock-stimulated control cells. It was described that the FOXP3 expression level in murine Tregs is correlated to their function [35]; however, in human Tregs, expression of FOXP3 is more complex, wherein human Tregs are known to express three different FOXP3 splice isoforms with functional consequences [8–11]. We therefore asked whether iTregs induced by the conditions under study, despite similar total FOXP3 protein levels on a per-cell basis, may show a change in FOXP3 isoform expression compared to nTregs. To this end and to further confirm the proteomics data with the additional aspect of FOXP3 isoform expression, we performed western blot analysis of iTregs and nTregs. These data confirmed higher FOXP3 protein expression in all iTreg populations compared to Mock-stimulated cells, although lower than in nTregs (Additional file 1: Figure S1e). The results further suggest that iTregs express functional FOXP3 isoforms with a similar isoform expression pattern as in nTregs (Additional file 1: Figure S1e). iTregs resembled the FOXP3+ ‘Treg’ subset of CD4+ cells based on FOXP3 and Eos expression, and also expressed low levels of cytokines ascribed to Th1, Th2, Th17, or Tr1 subsets such as IFN-γ, IL-4, IL-13, IL-17, IL-22, and IL-10 (Fig. 1c) (omics data in repository and [28]).

Together, these results demonstrate the quality of the iTreg cells used for molecular profiling based on canonical Treg ‘up’ signature molecules, such as the Treg markers FOXP3 and Eos, and low expression of Treg ‘down’ signature molecules such as IFN-γ.

### Time-course molecular profiling captures the dynamics and polarization of iTreg differentiation

We next performed a global analysis of the generated RNA-Seq and proteomics data from human iTregs and controls. In total, counting the GENCODE (v19) genes and applying a minimal filtering rule (≥ 1 count across all samples), we detected 33,991 genes. However, when considering an expression threshold (see Methods) for highly expressed genes (HEGs), which are more likely to be functional [36], our data comprised 15,910 HEGs corresponding to 11,378 (72%) protein-coding genes. With our mass spectrometry-based proteomics approach, we identified 9906 proteins (Ensembl protein ID), of which 6906 proteins (70%), corresponding to 6815 genes, were quantified in all samples, and accordingly used for relative quantification and statistical analysis. The overlap between the mass spectrometry-based proteome of 6815 genes and the HEGs was larger (55%) than the fraction of RNA-identified HEGs with no quantified protein (42%) (Additional file 1: Figure S2a, b). Despite the relatively high overlap, the number of detected features was higher for RNA-Seq than for proteomics, likely due to intrinsic technological features and not to major differences in the quality of the corresponding data. Indeed, we observed that the variability in both data types might primarily be explained by biological variables (such as the activation time or the treatment group) rather than technical variables, and that no major signs of unwanted batch effects were present (Additional file 1: Figure S3). This was also exemplified by the expected protein quantitative behavior, regardless of technical variables such as the Tandem Mass Tag (TMT) set, that was observed for known Treg proteins such as FOXP3 and Eos (Fig. 1e). Furthermore, we calculated the average number of proteins per cell [37], which included many important Treg and/or T cell regulatory proteins (Additional file 1: Figure S4), thus confirming the quality of our proteome data despite limited primary sample material.

**Fig. 2:**
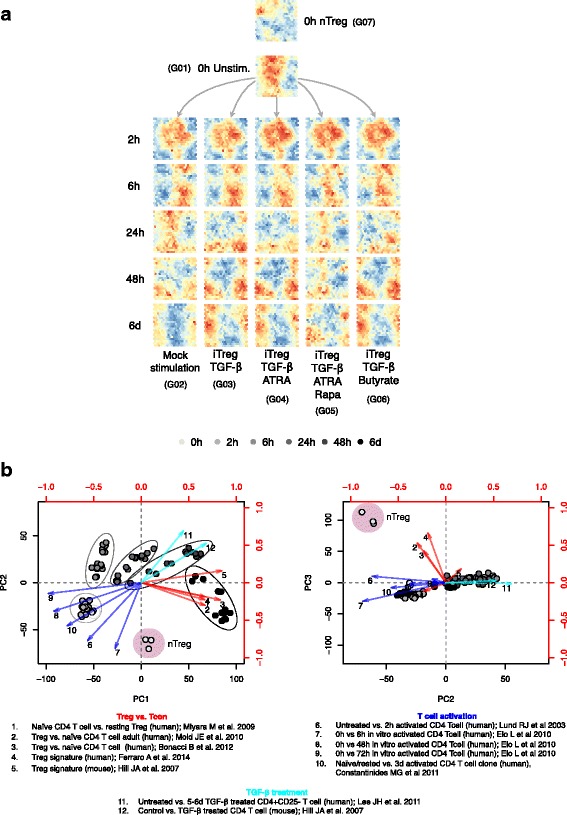
Global transcriptomic analysis gives a ‘bird’s-eye’ view of iTreg polarization and temporal gene expression architecture. **a** Self-organizing map (SOM) analysis of transcriptome data from Mock-stimulated (control) or iTreg cells induced by the indicated protocols shows the topology of the polarization at the transcript level. The panels are pseudo-colored SOMs from different time points (0, 2, 6, 24, and 48 h, and 6 days). The colors correspond to the average RNA expression levels (z-score) of the genes contained on each hexagonal cell (blue: low, red: high). The effect of the activation is predominant, but treatment differences become evident after 1 day of culture. **b** Principal component analysis differentiates the samples on the basis of the activation time and treatment; ellipses highlight samples from the same time point. Arrows correspond to selected gene signatures that correlate significantly with the first three principal components (PCs) (*p* < 10^− 6^ in either PC1, PC2 or PC3; Additional file 2: Table S1) and belonging to different functional categories (red: Treg vs. Tcon; blue: T cell activation; cyan: TGF-β treatment). PC scores are shown on the bottom and left axes, while top and right axes show the Pearson coefficient of each gene signature with the corresponding PC. The text on the bottom specifies the reference for each numbered signature (Additional file 2: Table S1)

To understand the dynamics of iTreg differentiation on a global scale, we relied on the wider coverage and sampling of the RNA-Seq data, while incorporating the effect of multiple experimental groups. Therefore, we performed unsupervised clustering using a version of Self-Organizing Maps (SOMs) that allows obtaining several parallel maps and perform data fusion (see Methods). As shown in Fig. 2a, the topology of the polarization indicated that a profound restructuring of gene expression was mainly observed over time for all conditions and already at the early time points, in line with the potent activation signals. Furthermore, when analyzed across conditions, the maps showed that differences were readily appreciable for the iTreg treatments compared to Mock stimulation, especially at late time points. When cross-comparing the iTreg time series, the TGF-β, TGF-β + ATRA, or TGF-β + butyrate conditions showed a similar overall molecular pattern, in contrast to TGF-β + ATRA + Rapa, which was one motivation to restrict the proteomics samples as well as further detailed analysis mainly to three treatment groups, namely Mock-stimulated cells, iTreg TGF-β, and iTreg TGF-β + ATRA + Rapa. Additionally, we have shown previously [28] that iTregs generated with TGF-β + ATRA + Rapa differed functionally, displaying enhanced suppressive activity in vitro.

Next, we asked whether the observed gene expression pattern over time was indicative of a bona fide iTreg polarization. As shown above, expression of *FOXP3* and *IKZF4* affirmed a Treg-like phenotype. To provide additional support and interpret the observed gene expression changes, we used publically available relevant T cell signatures. We obtained a three-dimensional map of the gene expression data using principle component analysis (PCA) (Fig. 2b and Additional file 1: Figure S3b); then, for each known signature and for each sample, we calculated a score, which essentially indicates whether a sample’s expression profile matches the known signature (Additional file 2: Table S1). The first two principal components (PCs) separated the cells mostly on the basis of the activation time and treatment group, the latter being more noticeable as time progresses and the spread increases (Additional file 1: Figure S3b, e). Correspondingly, when the signature vectors were correlated to the PC scores, PC1 and PC2 can be interpreted as related to cellular activation or the effect of TGF-β (selected ‘T cell activation’ and ‘TGF-β’ signatures in Fig. 2b). Interestingly, the ‘Treg vs. Tcon’ signatures were also positively correlated with PC1, suggesting that at least part of the variability observed over time may be correctly attributed to the anticipated Treg phenotype. Furthermore, inspecting the variation from the PC2–PC3 perspective, the in vitro polarized samples lie on a plane approximately defined by the activation and TGF-β signatures, while nTregs substantially diverge from this plane, and the human ‘Treg vs. Tcon’ signatures intersect this plane almost perpendicularly. While our analysis confirmed the similarity of the (unstimulated) nTreg samples with published Treg signatures (which, in the examples used, were based on unstimulated nTregs), it must be considered that the divergence of iTregs from nTregs may be largely influenced by the in vitro activation of iTregs, but may also reflect actual differences between iTregs and pTregs as well as between pTregs and tTregs or Treg subsets from different tissues [38–42]. Overall, the pattern of variability is readily compatible with a combined effect of time and experimental promotion of the iTreg phenotype as the driving forces shaping the differences between the samples.

### iTregs show protocol-specific and general patterns of differentially expressed genes and proteins

To model gene expression over time and call differentially expressed genes (DEGs), we specifically focused on the extraction of the genes that reacted in an iTreg-specific manner over time compared to the Mock-stimulated cells (iTreg group effect), as opposed to the genes that changed over time as a consequence of cellular activation (time effect). Given the different kinetics of general activation and correspondingly lower CD25+ cell fractions in iTregs induced with Rapa (Additional file 1: Figure S1c and Figure S3b, e), modeling gene expression over time and in comparison to Mock-stimulated control T cells was especially important. To ensure a robust calling of DEGs, we employed three different methods for differential analysis in parallel and required the genes to be significant for at least two methods (see Methods; Additional file 1: Figure S2c and Additional file 3: Table S2). Briefly, we either considered the time as a discrete or continuous covariate and performed appropriate multiple linear modeling with interactions between the time and the group. A corresponding analysis was done for proteomics data to obtain differentially expressed proteins (DEPs) (see Methods; Additional file 1: Figure S2d, e and Additional file 3: Table S2).

**Fig. 3:**
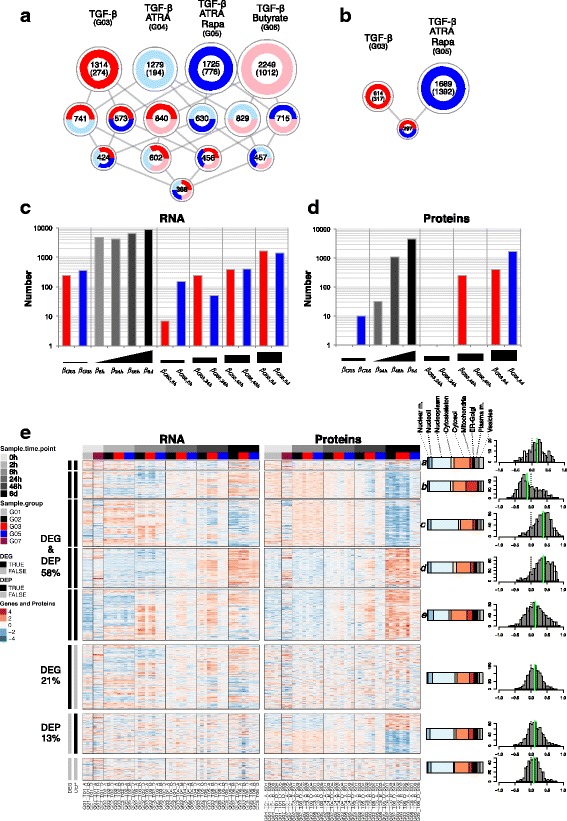
Differential gene and protein expression analysis during iTreg polarization. Differential expression was modeled over time (activation effect) and specific for iTreg induction (group effect). Group abbreviations: G01, Unstimulated naïve CD4+ T cells; G02, Mock-stimulated cells; G03, iTreg TGF-β; G04, iTreg TGF-β + ATRA; G05, iTreg TGF-β + ATRA + Rapa; G06, iTreg TGF-β + butyrate; G07, unstimulated nTreg. **a** DEGs in iTreg groups compared to Mock control in at least one time point or at baseline are counted. Number of DEGs in each condition or shared between iTreg conditions (see color code) is indicated and proportional to the circle size; numbers in parentheses are exclusive DEGs per condition. **b** DEPs in iTreg conditions compared to control are counted. Numbers of exclusive DEPs (in parentheses) or shared in the two iTreg conditions are shown. The number of (**c**) DEGs (FDR < 0.01) or (**d**) DEPs (FDR < 0.05) is shown for each of the indicated coefficients (grey-black: activation effect; red, blue: group effect) on a statistical model with time as a discrete factor. **e** A heatmap of RNA and protein data is shown for the genes detected at both levels. The expression is shown as separate RNA (regularized log (rlog)-transformed counts) or protein (log_2_R) z-score (blue: low; red: high). Black bars to the left indicate differential expression. Hierarchical clustering was performed separately for the indicated four blocks using RNA and protein data and clusters were obtained for the DEG and DEP blocks (see Methods). A, B, D: Donor 1, 2, 3; S01–S05: TMT set. Colored bars on the right show the relative fraction of the cellular compartments for the proteins with available data. Histograms show the distribution of the Spearman correlation; green line marks the median value

The massive cellular response to the activation was exemplified by the large number of genes (10,093 DEGs; 4660 DEPs) that responded to the stimulation over time (Additional file 1: Figure S2c, d and Additional file 3: Table S2). In addition to the general activation effect, a fraction of the genes responded with an iTreg-specific expression pattern, as iTregs presented 1279 to 2249 DEGs depending on the inducing protocol (Fig. 3a and Additional file 1: Figure S2c), of which several were shared between iTreg conditions (Fig. 3a). According to the stringent criteria used here to call DEGs, only 368 genes were DEGs shared between all four iTreg groups (Fig. 3a), and the iTreg TGF-β + ATRA + Rapa condition was bounding this number to a lower range when included in a two- or three-way comparison, in line with the SOM analysis (Fig. 2a) in which this group displayed a more unique profile, while the other iTreg groups were more similar to each other. Thus, for proteomics, iTreg TGF-β + ATRA + Rapa and iTreg TGF-β were used to represent the Treg-inducing protocols, and the following analyses are therefore focused on these two iTreg groups. Confirming the DEG data, almost 50% of the proteins differentially expressed in iTreg TGF-β were also DEP in iTreg TGF-β + ATRA + Rapa, while the latter had more unique differentially expressed molecules (Fig. 3a, b). Alongside protocol-specific differential gene and protein expression, we observed a shared signature induced by all the iTreg protocols, which was most relevant from the perspective of studying generic FOXP3-inducing molecules.

A special effort was made to separate the iTreg group effect from the relatively larger time effect (Fig. 3c, d), and the number of DEGs and DEPs for both the activation as well as the iTreg-specific effect increased over time, albeit with different kinetics for RNA and protein. More RNAs were already different at the first baseline point for RNA-Seq (2 h) but very few proteins were differentially expressed at the corresponding first available proteomics baseline point (6 h) and a similar number of DEGs and DEPs was only modeled at the terminal stage (Fig. 3c, d).

This global analysis was followed by a parallel comparison of RNA and matched protein expression over time. We asked whether a gene’s mRNA level correlated with its protein level, as translational efficiency, post-translational regulation, and molecular half-lives are known to affect the relationship between the two, especially during state transitions [43, 44]. The overlap of protein-coding DEGs with DEPs was 37% (Additional file 1: Figure S2e). However, since no corresponding protein was quantified for 61% of the protein-coding genes (Additional file 1: Figure S2a), we considered only RNA:protein pairs for which both data were available for further analysis. We observed that a substantial fraction (58%) of those genes was differentially expressed in both domains (Fig. 3e, DEG and DEP). Next, we assessed whether the changes in the two domains were correlated; to do so, we first partitioned the RNAs and proteins based on their corresponding differential expression and then further classified the largest block (DEG and DEP) into five clusters (see Methods and Fig. 3e). We observed that approximately half of the molecules in the DEG and DEP block had concordant expression (Fig. 3e, clusters *c*, *d*) and the other half belonged to clusters with discordant profiles. While the relative kinetics of the RNA and protein expression would allow for a delayed accumulation of the corresponding protein, resulting in a poorly correlated profile (as for cluster *e*), we observed one cluster of genes (cluster *b*) for which protein and RNA had instead an anti-correlated profile. Interestingly, this cluster was enriched for mitochondrial proteins and functions (Fig. 3e and Additional file 1: Figure S5). Similarly, for the other discordant cluster (*e*) more proteins were reported to localize in specific cellular compartments (nucleolus, ER/Golgi) rather than nucleus and cytoplasm, with corresponding matching functional enrichment (Fig. 3e and Additional file 1: Figure S5). This analysis demonstrates that, even when the transcriptional perturbations of a cellular system are accompanied by a corresponding substantial alteration of the proteome, the individual transcript level cannot be always considered a proxy for the corresponding protein. Therefore, when selecting genes for functional follow-up from transcriptome data, proteome data are an important non-redundant resource.

### iTreg gene expression signatures reflect specific functional programs

The functional transcriptional program of T cell activation and T helper cell subset differentiation in vitro [45] or T cell differentiation in the thymus [46] has been previously analyzed. Here, we specifically investigated the functional categories affected during the differentiation toward the iTreg subset, and we dissected, visualized, and grouped the distinct profiles of the genes that resulted differentially expressed as a function of the polarizing conditions (TGF-β or TGF-β + ATRA + Rapa).

**Fig. 4:**
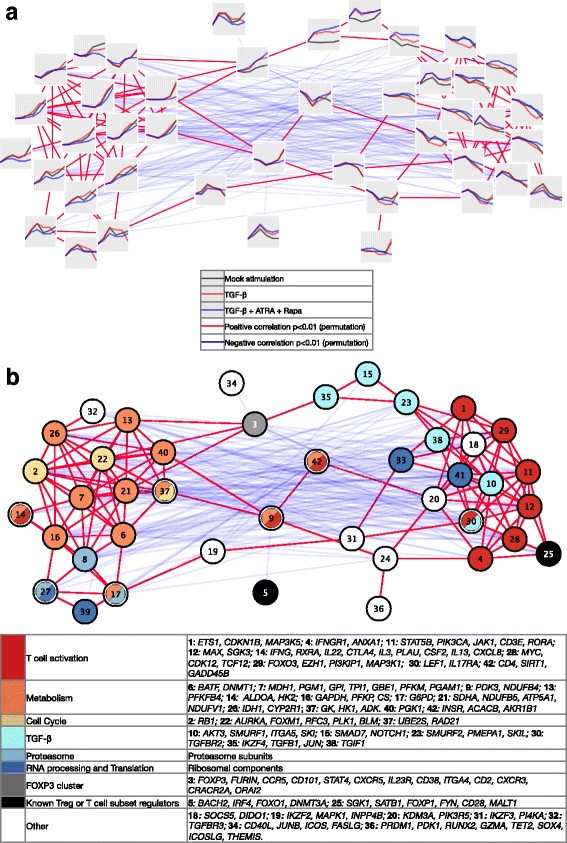
Clusters of genes show the functional dynamics of gene expression during iTreg differentiation. Model-based cluster analysis of gene expression reveals that the transcriptional profiles are functionally super-organized in concordant modules with functional similarity and show the molecular footprint of T cell activation and iTreg polarization. All DEGs in iTreg + TGF-β or iTreg + TGF-β + ATRA + Rapa compared to Mock-stimulated cells were considered. In (**a**), the average gene expression of the 42 clusters is shown after rlog transformation (x-axis: time; y-axis: average rlog-transformed RNA-Seq counts), with a colored line corresponding to the treatment group as shown in the legend. Each cluster is connected with a line to the clusters correlated positively (red) or negatively (blue), after permutation analysis (Spearman, *p* < 0.01). In (**b**), a functional category is assigned to the same clusters after gene ontology and pathway enrichment analysis. Bottom: the corresponding color legend and representative genes are given

To partition the genes with similar RNA expression over time, we used a model-based representation of the cluster of genes in the Mock control and in the iTreg time series (Additional file 4: Table S3). We further grouped the clusters using a summary measure of the correlation between the respective members (see Methods), and super-clusters were identified (Fig. 4a), i.e., two positively and densely connected components, one with a more generic proliferative and metabolic profile and another pointing to a more specific T cell program (Fig. 4a, b and Additional file 4: Table S3). The genes up-regulated over time were grouped at one extreme, while the down-regulated genes were found at the other. Moreover, for individual clusters, the differences between the iTreg and the Mock stimulation conditions were highlighted. The super-clusters not only showed data-driven dependence, but also sorted into interconnected domains with functionally related ontologies (Fig. 4b and Additional file 4: Table S3). Indeed, among the up-regulated clusters we identified enrichment for categories required and/or affected by a proliferative program, such as metabolism, cell cycle, RNA processing and translation, in line with the augmented metabolic requirements of activated T cells and a shift from a catabolic to an anabolic metabolic program [47]. We observed that iTregs showed differences in the cluster enriched for metabolic processes, especially for iTregs induced with TGF-β + ATRA + Rapa and in agreement with the metabolic control exerted on the in vitro induction of T cell subsets with specific requirements for Tregs, including a role for the mammalian target of rapamycin (mTOR) [12, 31]. At the other extreme of the cluster network, a specific effect of the iTreg polarization conditions was shown for the clusters identified as ‘TGF-β clusters’ (clusters 10, 15, 23, 35, 38), mostly containing genes that were more abundantly expressed in the iTreg as compared to the Mock control time series (e.g., *IKZF4*, *SMAD7*, *NOTCH1*, *TGFB1*, *SKIL*). In the proximity, other ‘T cell activation’ genes (clusters 1, 4, 11, 12, 28, 29) were instead abundantly expressed at the beginning of the polarization but down-regulated over time (e.g., *MAP3K1*, *STAT5B*, *MYC*, *MAX*, *CD3E*). Interestingly, the *FOXP3*-containing cluster 3 appeared as being central in the network of clusters and bridging between the TGF-β series of clusters and the metabolism/cell cycle groups (clusters 13, 21, and 22). *FOXP3*, *FURIN*, *CCR5*, *CD101*, *CD2*, and *CXCR5* were among the cluster 3 members and concordantly upregulated in iTregs. In summary, the functional gene expression program agrees with simultaneous proliferation, activation, and polarization, a typical behavior of differentiating T cells.

### iTreg gene expression program inferred from network reconstruction

**Fig. 5:**
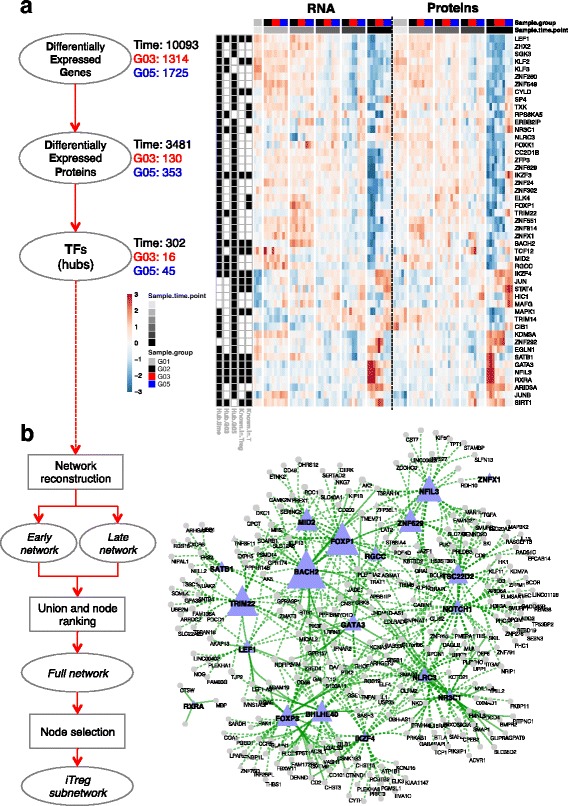
Treg factors represent hubs in a core network of known and novel genes with regulatory potential. A hub-centered approach was employed to reconstruct a gene co-expression network and the temporal rewiring of the nodes. **a** Hubs were defined as TFs which were DEGs and DEPs (see Methods). Numbers correspond to the counts of selected features at each step. A heatmap shows the relative gene (rlog counts) and protein (log_2_R) expression (z-score) for the hubs differentially expressed in iTregs (G03: TGF-β and/or G05: TGF-β + ATRA + Rapa). Abbreviations and color codes as in Fig. 3. Black boxes to the left of the heatmap indicate whether the given TF is differentially expressed over time (‘hub.time’) or in iTregs (‘Hub.G03’ or ‘Hub.G05’) in at least one time point; the gene has a known role in T cells or specifically Tregs (‘Known.in.T’ or ‘Known.in.Treg’). **b** Two networks were reverse-engineered using the early or late time point samples, then, a rewiring score was calculated for each node by comparing them (see Methods, Additional file 1: Figure S6b, c, Additional file 5: Table S4). Shown is the sub-network of nodes that were modeled as DEGs in all four iTreg conditions, in addition to FOXP3. A triangle marks TFs. Light blue nodes correspond to the hubs and their size is proportional to the rewiring score. A green continuous line marks the edges with support from a TF:target gene network built from ENCODE data (see Methods). Unconnected nodes are not displayed

The polarization of iTregs from naïve T cells over time may encompass regulatory interactions that are changing over time as a result of the dynamic evolution of the system. Indeed, we showed above (Fig. 4) that the expression profile of the gene cluster comprising *FOXP3* did not positively correlate with the profile of the clusters containing known *FOXP3* activators (e.g., *BACH2*, *FOXO1*, *STAT5B*, *FOXO3*). The relative expression over time of each member of a regulator:target pair is key in determining the biological outcome and a simple approach based on correlation does not incorporate such a dynamic interaction. Therefore, we hypothesized that, after considering the temporal factor in reconstructing the network of interacting genes, it would be possible to retrieve the known, and possibly novel, molecular connections from our data and that we could apply the ‘guilty by association’ principle in order to retrieve original candidate regulatory genes. The most common dynamic network reconstruction algorithms require a very dense sampling rate to estimate lagged dependencies and the small sample size might be a limiting factor. We therefore sought to overcome this limitation by comparing the gene networks reconstructed statically but for two sets of samples, i.e., the ‘early’ (0–6 h) and the ‘late’ (24 h to 6 days) observations independently, and capture the temporal aspect of the regulatory system by inspecting the rewiring of the nodes. Indeed, when summarizing the expression profile of known *FOXP3* positive transcriptional regulators as a signature score (see Methods; Additional file 1: Figure S6a), we verified that there is an early bout of induction of those regulators peaking at 6 h, except for *RUNX1*, *NFATc4*, and *FOXP3* itself, which were upregulated later and specifically in iTregs. These observations are consistent with an accumulation of *FOXP3* at later time points. We implemented a hub-centered approach to infer the connections between the genes in the early and late stages of the differentiation using a mutual information (MI) criterion and calculated a rewiring score by comparing the two networks (Additional file 5: Table S4). We robustly selected the hubs as the set of 307 TFs resulting differentially expressed both at the protein and RNA level after cellular activation or iTreg polarization. Of these, the subset of 49 TFs differentially regulated in iTregs (Fig. 5a) includes genes with a well-known role in Treg biology, which are maximally transcribed either early (*BACH2*, *LEF1*, *IKFZ3*, *SATB1*) or late (*IKFZ4*, *NFIL3*, *GATA3*, *JUN*, *RXRA*) in our system and show generally concordant RNA and proteins levels, in addition to novel factors not known in Treg/T cells (Fig. 5a). We obtained bootstrapped consensus early and late networks by calculating the MI between the hubs and all the other expressed genes. The two consensus networks were combined by union to derive a *full network* and a node-rewiring score [48] was used for ranking (see Methods and Additional file 1: Figure S6b, c). A major rewiring force was the T cell activation through CD3, CD28, and IL-2, which constitute a well-known potent signal for the dynamics of the cellular network. Therefore, we focused on the specific events occurring in iTregs by selecting a subnetwork formed by FOXP3 and the shared DEGs in all iTreg conditions (Fig. 3a), here called iTreg subnetwork. The term ‘iTreg subnetwork’ was chosen to indicate the differential expression in iTregs; however, it should be noted that several of these genes also appear similarly expressed in nTregs (Additional file 1: Figure S6d). This selection resulted in a total of 349 nodes (Fig. 5b), of which 19 were TF hubs. Notably, although the MI provides only an undirected graph, taking the direction from the TF hub to the target node into account, a direct comparison with literature and external data sources can be made in order to verify previously observed regulatory interactions. Indeed, several connections in our network were supported by a network of TF binding, independently reconstructed from DNase-Seq data from Tregs (see Methods; Fig. 5b). Such comparison, however, is limited due to incompleteness of the binding motif database. The selected iTreg subnetwork (Fig. 5b) was enriched for genes of the TGF-β pathway, as indicated by functional enrichment analysis using Gene Ontology, KEGG, Reactome, and MSigDB Immunological Signatures databases (Additional file 6: Table S5) and as expected by the involvement of TGF-β signaling in iTreg differentiation [16]. We next explored whether the iTreg subnetwork genes may be differentially expressed simply as a consequence of FOXP3 expression in iTregs. Time-course analysis of gene expression profiles revealed that the majority of the iTreg subnetwork genes displayed differential expression prior to FOXP3 expression, suggesting that they may play a role in FOXP3 induction (Additional file 1: Figure S6d, e). Further, many iTreg subnetwork genes were represented in those gene clusters functionally assigned to TGF-β signaling earlier (Fig. 4), suggesting that they may be involved in the TGF-β pathway and not indirectly regulated by FOXP3 (Additional file 1: Figure S6e). Nevertheless, several iTreg subnetwork genes displayed a similar temporal expression profile as FOXP3 (Additional file 1: Figure S6d), and thus it is possible that they may be regulated by FOXP3 itself. To further explore this possibility, we studied the expression of iTreg subnetwork genes in published data [49] of pre-activated primary human naïve CD4+ T cells upon FOXP3 transduction compared to an empty vector. We determined that 9 of 349 iTreg subnetwork genes (including FOXP3) were significantly (false discover rate (FDR) < 0.05) differentially expressed in FOXP3- versus control-transduced cells (Additional file 1: Figure S6f), suggesting the possibility that these few genes may be expressed as a consequence of FOXP3 in iTregs.

In conclusion, our approach defined a novel iTreg subnetwork containing multiple genes potentially involved in FOXP3 induction. Importantly, along with novel potential Treg regulators, the iTreg subnetwork revealed known crucial Treg regulators like *BACH2*, *NFIL3* (E4BP4), *GATA3*, *NOTCH1*, *LEF1*, *SATB1*, and *IKZF4* (Eos) with a well-known role not only in iTregs, but also, and more importantly, in vivo in nTregs (Fig. 5a, b).

### The selected iTreg subnetwork contains autoimmune disease-associated genes

The immunomodulatory and tolerogenic activities of Tregs in vivo are well-known and exemplified by the severe human disease immunodysregulation polyendocrinopathy enteropathy X-linked syndrome, which is caused by FOXP3 mutations [4] as well as by the contribution of Treg defects to multiple complex human autoimmune diseases [50]. Based on the therapeutic activity of Tregs in numerous pre-clinical mouse models of autoimmune and inflammatory diseases in the past, recent advances have been made towards clinical trials applying Treg therapy, primarily in GvHD, transplantation, and T1D [12, 13]. For these reasons, we hypothesized that the genes that are affected by our experimental iTreg induction may be relevant in disease pathways.

**Fig. 6:**
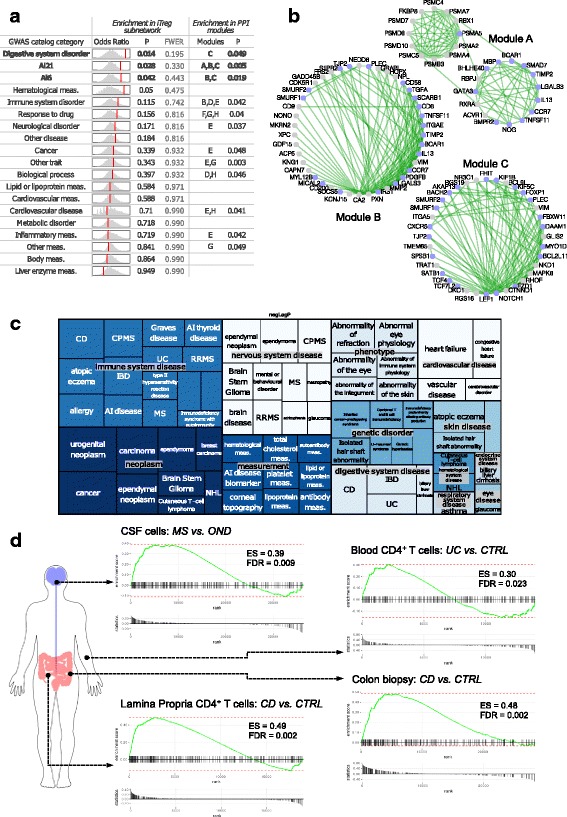
The iTreg subnetwork is linked to common autoimmune diseases. The selected iTreg subnetwork (see Fig. 5) was tested for connection with diseases using multiple public sources of annotation. **a**, **b** The enrichment of the GWAS catalog disease categories in addition to two categories of autoimmune diseases (Ai6 and Ai21, see Results text) is shown. For each category, the null distribution of the odds ratios (ORs) obtained with 10,000 random gene sets from the full network is shown as a histogram and the observed OR with a vertical red line. The categories in bold have a resampling-based *p* value of < 0.05. The resampled OR null distribution was used to calculate a FWER using the step-down minimum *p* value procedure. Enrichment in PPI modules is shown for categories with a nominal *p* value of < 0.05 (Fisher’s exact test), and 119 hypotheses were tested. The modules from a PPI network are given for the corresponding enriched categories and three PPI modules associated to the top three enriched in iTreg subnetwork disease categories are displayed in (**b**). Light blue nodes correspond to genes with an associated SNP in the GWAS catalog. **c** The treemap shows the enrichment *p* values of the indicated Open Target categories for the iTreg subnetwork. Only disease associations with nominal *p* < 0.05 are shown. The size of the square is proportional to the –log_10_(p) and disease types are grouped by therapeutic areas. **d** Gene Set Enrichment Analysis (see Methods) confirms that the iTreg subnetwork gene set is positively enriched in the ranked list obtained when comparing gene expression in CD4+ T cells from gastrointestinal tissue or blood of inflammatory bowel disease patients (UC or CD as indicated) or from cerebrospinal fluid (CSF) of MS patients compared to controls. CTRL: control, symptomatic or healthy; OND: other neurological disease; ES: enrichment score

Firstly, we tested whether any disease categories were enriched in our iTreg subnetwork as compared to the full network. Thus, we tested the genome-wide association studies (GWAS) catalog terms grouped by their disease ontology plus the additional Ai6 and Ai21 categories, which respectively include the GWAS genes for six common autoimmune diseases (multiple sclerosis (MS), rheumatoid arthritis, T1D, Crohn’s disease (CD), systemic lupus erythematous, and psoriasis) or the 21 autoimmune disorders as in Farh et al. [51]. We observed notable overlap for autoimmune diseases as compared with 10,000 random gene sets selected from the full network (Ai6: odds ratio (OR) 1.57, *p* = 0.042, family-wise error rate (FWER) 0.443; Ai21: OR 1.55, *p* = 0.028, FWER 0.330) (Fig. 6a and Additional file 6: Table S5), and therefore corresponding to genes expressed in CD4+ T cells during activation/differentiation. We also used the association as in Menche et al. [52] as another catalog of diseases genes, further showing that disorders of the digestive and nervous system with an autoimmune component were ranked at the top of the enriched diseases (Additional file 6: Table S5). Indeed, when we compared the iTreg subnetwork to the mapped genes for CD in the GWAS catalog, we retrieved genes with variants previously identified in GWAS scans (*ZFP36L1*, *PLCL1 DENND1B*, *BACH2*, *SLC22A23*, *JAZF1*, *IL18RAP*, *NOTCH1*, *TSPAN14*, *CD6*). Similarly, MS susceptibility genes (*CD6*, *CXCR5*, *ZFP36L1*, *KIF1B*, *CD58*, *BACH2*) appeared in the iTreg subnetwork and we detected a positive enrichment for MS.

Secondly, as it has been shown that a network approach based on protein–protein interactions (PPI) can guide the interpretation of GWAS data [52], we identified the modules on a reference PPI network that were enriched for the above disease categories (see Methods). For three PPI modules (out of 119 modules) we detected an overrepresentation of the Ai6, Ai21, and/or digestive system disorder categories (Fig. 6a, b), further supporting the hypothesis that the iTreg subnetwork contains genes that act in the same autoimmune disease neighborhood, such as *LEF1*, *BACH2*, *TCF4*, *KIF1B*, *CXCR5*, and *NOTCH1*, which have been implicated in autoimmune diseases by independent reports.

Thirdly, to further support the connection with autoimmunity, we interrogated the Target Validation Platform of the Open Targets Consortium (www.targetvalidation.org) and retrieved all the association scores, which summarize the association evidence between a drug target and a disease, and it is calculated from various data sources, including genetic associations, somatic mutations, known drugs, pathways affected by pathogenic mutations, RNA expression, animal models, and text mining. When testing the diseases associated with the iTreg subnetwork as compared to the full network, we grouped the diseases with a nominal *p* value of < 0.05 (hypergeometric test) by therapeutic area and again verified that ‘Immune system disease’ was the prominent therapeutic area, including several autoimmune diseases such as MS and inflammatory bowel disease (IBD, including both CD and ulcerative colitis; Fig. 6c), further confirming that the selection of iTreg genes is an appropriate criterion for identifying known and likely also novel genes with relevance for human autoimmune diseases.

Given the above findings based mainly on genetic susceptibility, we formulated the hypothesis that, in the context of the diseases for which there is a likely contribution of T cells and in particular Tregs, the iTreg subnetwork genes should positively overlap with the genes whose expression is deregulated in patients compared to controls. Therefore, we retrieved publicly available gene expression signatures (see Methods) for IBD and MS – diseases in which Tregs are known to be relevant – and performed a Gene Set Enrichment Analysis with the iTreg subnetwork gene set. A positive enrichment was indeed observed when using the ranked signatures obtained from CD4+ T cells, not only from the affected tissue, but also from blood (Fig. 6d).

### Molecular profiling reveals novel candidate molecules that can classify iTregs versus Mock-stimulated T cells

**Fig. 7:**
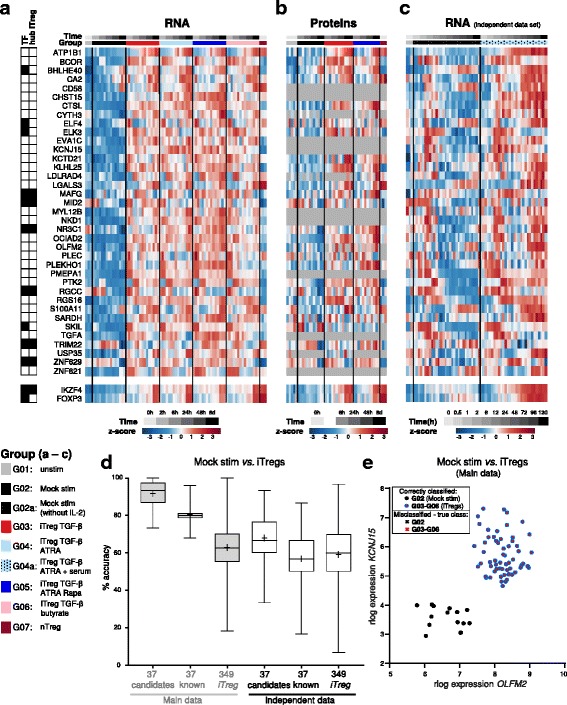
Molecular profiling reveals known and novel Treg regulators. Novel ‘candidate’ molecules putatively involved in FOXP3 induction were selected, along with ‘known’ Treg regulators as control (see text and Additional file 1: Figure S7a, b). **a**–**c** Expression profile of novel iTreg candidate molecules. *IKZF4* (Eos) and *FOXP3* are shown for comparison. Labels as in Figs. 1 and 5. **a** Candidate gene mRNA counts were rlog-transformed, and row-normalized z-scores are displayed (blue: low, red: high expression). **b** iTreg candidate protein expression (log_2_R). Grey cells: the protein was not detected in the respective sample. **c** iTreg candidate gene expression from an additional, completely independent RNA-Seq dataset is displayed and analyzed as in (**a**). iTregs were cultured with TGF-β + ATRA + serum, and additional time points were measured. **d**, **e** In silico validation of novel candidate molecules for iTreg classification. **d** Linear discriminant analysis (LDA) with all possible combinations of two genes out of ‘37 candidates’, ‘37 known’ Treg, or 349 nodes in the iTreg subnetwork (‘349 iTreg’) lists, followed by group classification. Grey boxplots show the cross-validated accuracy of classifiers regarding discrimination of Mock-stimulated (G02) vs. iTregs generated with either protocol (G03–G06) in the Main dataset. White boxplots show results from LDA analyses performed in the same way, but for discrimination of Mock-stimulated cells (G02a) vs. iTregs (G04a) in the independent dataset. Whiskers: min. to max. Value; + mean value; *n* = 1332, 1332 and 121,452 pairs for ‘37 candidates’, ‘37 known’, and ‘349 iTreg’, respectively. The adjusted *p* value was ≤ 0.0001 by Kruskal–Wallis test with Dunn’s multiple comparison test for each vs. each boxplot. **e** Example of two candidate genes (top classifiers) which separate Mock cells from iTregs in the Main dataset with 100% accuracy (0% error) in LDA

The above analyses confirm that the iTreg data presented here revealed disease-relevant and known important Treg regulatory genes. We therefore selected novel ‘candidate genes’ that appeared likely to have a role in iTreg generation, and specifically FOXP3 induction, for further investigation. Those ‘candidate genes’ (Fig. 7a and Additional file 1: Figure S7a) were chosen based on satisfying at least two of the following criteria: (1) being shared DEGs in all four iTreg conditions (Fig. 3a); (2) being nodes in the iTreg subnetwork (Fig. 5b); (3) integrating DEGs and DEPs in iTregs versus Mock control cells and sub-setting on TFs (‘iTreg hubs’ in Fig. 5a); or (4) for these genes, a similar expression profile on protein level if available. Based on integrating these criteria and ascertaining, through a literature search, that a role in Tregs had not been elucidated at the time, 37 novel ‘candidate genes’ with a putative role in FOXP3 induction were chosen. We preferably selected putative positive regulators of FOXP3 upregulated in iTregs compared to Mock-stimulated cells (Fig. 7a, b) and, thus, amenable to perturbation approaches by gene knockdown. Notably, the candidate genes chosen here were over-represented in few gene clusters according to Fig. 4 (Additional file 4: Table S3), that is, clusters 3 (‘FOXP3 cluster’), 5 (‘known regulator’ cluster), and 23 and 35 (‘TGF-β clusters’).

Besides the ‘Main dataset’ described above, we also provide another unpublished, completely independent additional RNA-Seq dataset (here called ‘independent RNA-Seq dataset’). This independent dataset comprises Mock-stimulated cells and TGF-β + ATRA iTregs induced under slightly different culture conditions (see Methods), from different donors and laboratories, and including additional time points. A high fraction of FOXP3-expressing cells in the population, as well as the suppressive activity of these iTregs, was confirmed (Additional file 1: Figure S7c, d). Further, early and stable *IKZF4* mRNA expression as well as high *FOXP3* mRNA in iTregs compared to Mock-stimulated cells was confirmed in these independent donors (Fig. 7c). Importantly, the fractions of activated (CD25+) cells in iTregs and Mock-stimulated cells were high and in a similar range between these conditions (Additional file 1: Figure S7c), allowing accurate comparison of gene expression in these cells even with bulk populations. When studying the expression pattern of the above-selected candidate genes in this independent dataset (Fig. 7c), we observed that most candidates showed a similar expression pattern in both datasets, confirming the general trend of iTreg-specific candidate gene expression as compared to Mock-stimulated cells (Fig. 7a–c). Additionally, the extended time-series revealed an early spike of expression for several candidate genes and a more clearly divergent expression between iTregs and Mock-stimulated cells at later time points of differentiation (Fig. 7c). Additionally, from a global perspective, PCA incorporating the shared treatment groups and time points showed that both the Main and independent dataset displayed a similar behavior, namely the time (activation effect) predominantly explained the variability and, further, a separation of the Mock control and iTreg groups was increasingly evident as time progressed (Additional file 1: Figure S7e).

We next performed an ‘in silico validation’ to test whether the newly defined candidates could separate iTregs from other cells in a better way than known Treg regulators or genes randomly chosen from the iTreg subnetwork nodes. Both candidate genes and known Treg regulators (Additional file 1: Figure S7a, b) were retrieved from the list of genes being DEGs in at least one iTreg condition compared to the Mock stimulation control (Fig. 3a). We first tested the classification capabilities on the Main RNA-Seq dataset that was used to identify the candidate genes, although the obtained accuracy estimates are known to be optimistically biased (see [53] and below). We used a Linear Discriminant Analysis (LDA) approach [54] followed by group classification and, as expected, the candidate genes performed better than known Treg regulators (or genes from all the 349 nodes in the iTreg subnetwork) in classifying iTregs (induced by any protocol) compared to Mock-stimulated cells (Fig. 7d). In detail, several single candidates were able to classify iTreg versus Mock cells with > 94% accuracy (data not shown). Similarly, many combinations of two candidates could classify iTreg versus Mock cells with 100% accuracy, while the maximal cross-validated accuracy for pairs from the 37 known Treg regulator list (including *FOXP3* and *IKZF4*) was 96% (Fig. 7d, e).

Because the novel iTreg candidate gene set was created using all the Main dataset, the accuracies reported by the above LDA analyses are subject to the well-known feature selection bias [53]. For example, the selected candidates (and known regulators) were differentially expressed in iTregs in the Main dataset, and also the same dataset was used for group classification in LDA. Moreover, most of the candidate genes (35/37) and known regulators are represented within the iTreg subnetwork control gene list, largely explaining the high classification accuracy for some pairs in the control list (Fig. 7d). To further confirm the classification ability of the candidate genes, we therefore performed additional analyses as indicated below.

Importantly, we confirmed the classification power of the candidate molecules in the independent RNA-Seq dataset that was not used for candidate selection. To do so, we tested all possible gene pairs within all three lists (37 candidates, 37 known, 349 iTreg) as above with LDA in the independent dataset. In this independent dataset, we could separate iTregs from Mock-stimulated T cells using candidate gene pairs with up to 93.3% cross-validated accuracy (mean ± SD 67.97 ± 10.63%), which was significantly higher (*p* < 0.0001) than with pairs from the 37 known Treg regulators or the 349 iTreg subnetwork gene lists (mean ± SD 56.8 ± 12.1% and 59.1 ± 13.8%, respectively; white boxes in Fig. 7d). To further explore good classifiers, we also sub-selected all 76 candidate gene pairs that performed with 100% accuracy in the Main dataset. This set of ‘top classifier’ pairs included all 37 candidate molecules, though some occurred more frequently than others (Additional file 1: Figure S8a). These top classifier pairs were able to separate iTregs and Mock cells in the independent dataset with up to 90% accuracy (mean ± SD 72.1 ± 7.7%; Additional file 1: Figure S8a–c).

As a second approach to confirm the iTreg classification ability of the candidate genes without feature selection bias, we used a Random Forest (RF) classification using all 15,910 HEGs as input to rank the individual genes for their ability to distinguish iTregs from Mock cells (Additional file 7: Table S6). Importantly, 21 (57%) of the candidate genes and only 2 genes of the 37 known Treg regulator list were represented in the top-ranking 100 genes (Additional file 1: Figure S8d and Additional file 7: Table S6), confirming the candidate genes’ individual ability for distinguishing iTregs from Mock cells with this unbiased RF analysis. Interestingly, among the top-ranking 100 genes, we observed several of the genes that also occurred frequently in top classifier gene pairs with 100% accuracy in the above LDA analysis, namely *OCIAD2*, *OLFM2*, *CTSL*, *NKD1*, *TGFA*, *KCNJ15*, *SARDH*, and *PMEPA1* (Additional file 1: Figure S8a, d and Additional file 7: Table S6)*.* Regarding important known Treg regulators, *FOXP3* ranked at position 101, *IKZF4* at position 5, and *FURIN* at position 8. Notably, half of the top-ranking 20 genes were candidate genes, among other molecules (Additional file 1: Figure S8d). Nevertheless, we did not prioritize the latter for follow-up analyses as several of these genes are not protein-coding or their role in Tregs or other T cells is already well known.

Together, these data confirm that the selection of candidate molecules was better than a set of known Treg regulators in classifying iTregs in the Main dataset as well as in an independent dataset.

### Experimental validation of novel candidate molecules confirms functional effects on FOXP3 expression

The above analyses confirmed that several candidate molecules performed well in distinguishing Mock-stimulated cells from iTregs; however, differential expression does not necessarily mean that these molecules are involved in iTreg biology and FOXP3 induction. Therefore, we sought to validate the putative functional role of the candidate molecules in FOXP3 induction through a targeted shRNA screen. We produced lentiviral particles delivering shRNA pools against each of the candidate molecules (Additional file 8: Table S7), side-by-side with control lentiviruses (shScr or empty vector as negative controls, shFOXP3 or shIKZF4 as positive controls) and transduced primary T cells, which were then differentiated under iTreg or Mock stimulation conditions. To maximize knockdown efficiency in our screening setup, we used pools of shRNAs and, when possible, validated clones from the TRC library (Additional file 8: Table S7), although off-target effects cannot be excluded in such a screening setup. Efficiency of pooled shRNA versus single shRNA was tested beforehand using shRNAs against CD4 and measurement of CD4 knockdown efficiency (Additional file 1: Figure S9a).

**Fig. 8:**
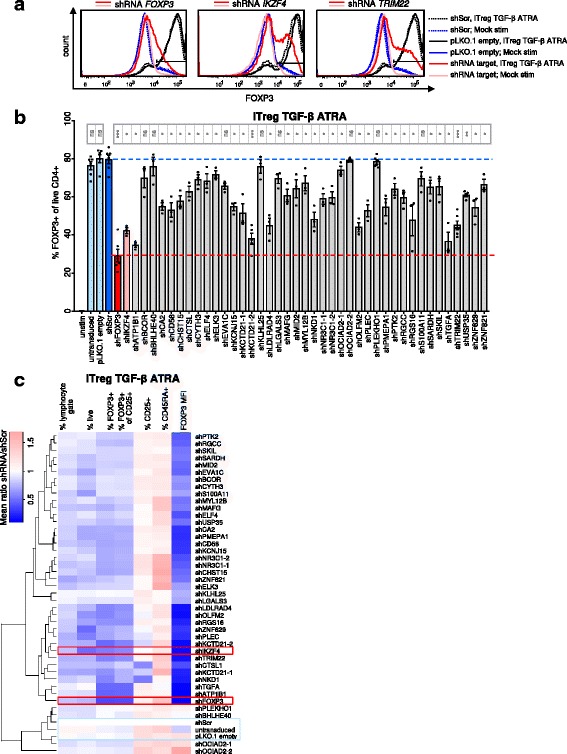
Experimental validation of novel candidate molecules regulating FOXP3+ Tregs. **a**–**c** 37 novel candidate genes with a putative role in Treg induction were chosen for a targeted shRNA validation screen. Transduced or ‘untransduced’ primary CD4+ T cells were cultured under iTreg conditions (TGF-β + ATRA), or left unstimulated (‘unstim’), and then stained for FOXP3 and other markers. **a** FOXP3 histograms for T cells transduced with shRNA targeting *FOXP3*, *IKZF4*, or the candidate gene *TRIM22* (red lines). Black and blue lines: negative control shRNA (shScr), empty vector (pLKO.1 empty). A representative donor of 3 (*IKZF4*) or 6 (*FOXP3*, *TRIM22*) is shown. **b** FOXP3 expression (gated on live CD4+ cells) in iTregs transduced with shRNA targeting candidate genes (grey bars; ‘–1’ and ‘–2’ indicate two independent shRNA pools). Blue, red bars: negative, positive controls. Displayed are mean ± SEM values, each dot represents an individual T cell donor (*n* = 3–6 donors from two independent experiments). FDR-adjusted *p* values (two-sided *t* test shRNA vs. shScr paired within a donor) are labeled as follows: ns *p* > 0.05; * *p* < 0.05; ** *p* < 0.01; *** *p* < 0.001; **** *p* < 0.0001. **c** FOXP3 and other flow cytometry markers in shRNA-transduced iTregs as in (b). The ratio of each marker relative to shScr-transduced cells of the same donor was calculated, and mean values of *n* = 3–6 donors are indicated by the color scale. It was pre-gated on live lymphocytes except for % lymphocyte gate and % live parameters. *MFI* median fluorescence intensity. Hierarchical clustering: complete linkage based on Euclidian distance

Transduction efficiency, as measured by GFP transduction, was approximately 50–65% (Additional file 1: Figure S9b) and transduced cells were selected by puromycin during differentiation, following which FOXP3 and other markers were measured by flow cytometry. Targeting FOXP3 directly with shRNA led to almost complete knockdown of FOXP3 expression, and several negative controls (untransduced, pLKO.1 empty, and shScr vectors) did not affect FOXP3 induction, confirming absence of unspecific effects due to the procedure (Fig. 8a, b). As a result of our candidate validation screen, we found that shRNA targeting of most (30/37; 81%) of the candidate molecules resulted in significantly lower fractions of FOXP3+ cells in iTreg cultures (Fig. 8a–c). This screening setup suggests a functional role for these 30 candidates in FOXP3 expression; nevertheless, it should be noted that future studies are needed to confirm these results for individual candidates and in different experimental approaches in more detail. Some candidate molecules not only affected FOXP3 but also the general activation of T cells, as measured by CD25 upregulation and CD45RA downregulation (Fig. 8c). For several candidates, activation-induced, low-level FOXP3 expression in Mock-stimulated cells was also affected in cells transduced with targeting shRNA, albeit with fewer significant hits (Additional file 1: Figure S9c, d). Another indication for a biological relevance of the novel candidate molecules in Tregs could be their alteration in immune diseases with known Treg involvement. Notably, several candidate molecules scored as being associated with immune diseases, including MS and IBD (Additional file 1: Figure S7a).

Together, our data provide a resource of high-quality mRNA and protein data obtained at multiple time points during the differentiation of human primary iTregs induced under several different conditions and across different laboratories, along with Mock-stimulated T cells, unstimulated naïve CD4+ T cells, and CD25^high^ nTregs. Our results uncovered a range of known crucial Treg regulators with relevance to several immune diseases such as IBD, along with several novel factors that are likely to play equally important roles in FOXP3 expression. Indeed, our in silico and experimental validation confirmed that the majority of the carefully selected novel candidate Treg regulatory molecules successfully classified iTregs and that their experimental perturbation led to reduced induction of FOXP3-expressing T cells.

## Discussion

Herein, we provide a resource of RNA-Seq and proteomics data covering a time-series during human iTreg differentiation, along with control activated, non-activated, and nTreg cells. To our knowledge, this is the first report of unpublished iTreg proteome data to date. Although the transcriptional signature of murine and human TGF-β and TGF-β + ATRA iTregs has been analyzed [3, 38, 39, 55, 56], transcriptional profiles of iTregs induced by the additional protocols used here have not been studied. Profiling iTregs induced with several protocols in parallel allows for the detection of generic, protocol-independent FOXP3 inducers and at the same time reveals information about specific Treg signatures induced by certain compounds. For example, ATRA has a well-known induction effect of gut-homing properties [17] and Rapa inhibits the Akt/mTOR pathway and downstream genes [30]. Furthermore, previous studies on murine iTregs did not include time-series but only end-stage transcriptional analysis of fully differentiated cells, while the driving molecules involved in initiation of the differentiation program and FOXP3 upregulation likely operate earlier and might be entirely missed with these approaches. One recent study presents time-series data during human Treg induction with TGF-β [55]; however, the authors did not further focus on Tregs and no significant enrichment for disease-associated SNPs in TF genes was found in iTregs in their study. This is contrary to our study, in which we detect an association of iTreg genes with several immune system diseases, although it should be noted that the results from our disease enrichment analyses must be regarded solely as a comparative relative enrichment of certain diseases over others, complicated by the fact that the disease categories are not independent and therefore standard methods for *p* value correction are likely too conservative. Further, the studies may not be absolutely comparable because, in the aforementioned study [55], FOXP3 was neither measured on protein nor at the single-cell level and iTregs expressed relatively low *FOXP3* and relatively high *TBX21* and *RORC* mRNA compared to Th1 and Th17 cells, respectively, potentially suggesting sub-optimal iTreg differentiation. In contrast, the transcriptional data in the present work comprises experiments from two different laboratories, in both of which the iTreg phenotype was assessed with Treg markers, such as the expression of FOXP3 protein at the single-cell level, and the suppressive function was tested. Furthermore, we present matched RNA and protein expression of other Treg signature genes such as the important Treg regulator Eos [33], which is not usually measured in human Tregs due to limitations for detection by antibody-based methods. Interestingly, iTregs induced by our protocols expressed high amounts of Eos protein and mRNA (reaching levels similar to nTregs), a feature that was previously shown to require more than simple ectopic FOXP3 expression, suggesting an association with an nTreg-like phenotype [26, 57]. We confirmed high expression of Eos protein in human nTregs and, importantly, our unique time-course iTreg transcriptome and proteome data show that Eos was one of the strongest and earliest upregulated molecules in all iTreg conditions tested. Therefore, we included Eos as a further positive control for shRNA validation and we showed, for the first time, that knockdown of Eos strongly reduced FOXP3 expression in human iTregs.

In our study, the availability of time-series RNA-Seq data along with proteomic data and an additional independent RNA-Seq experiment with similar molecular patterns enabled the exploration of a most robust iTreg signature and the containment of differences due to experimental setup or technical factors. Nevertheless, it should be noted that limitations still exist, including individual donor variability and the number of biological replicates and time points available. Another important aspect to consider when using this data resource for future studies is the different fraction of activated (CD25+) and FOXP3+ cells under diverse differentiation conditions. Measuring gene expression in these heterogeneous bulk populations may affect calling certain differentially expressed genes, and single-cell analyses or studies in sorted sub-populations may enable identification of additional factors in the future – although these methods are not without their own limitations. Since we were interested in early events preceding FOXP3 induction, and CD25 as a general activation marker was upregulated only after 12–24 h, it was not possible to perform the expression profiling on re-sorted CD25+ cells. Furthermore, sorting would take time and thus affect the accuracy of the time points, but more importantly the global T cell transcriptome is sensitive to changes induced by magnetic or flow sorting [58, 59]. Therefore, we freshly isolated the cells from peripheral blood, rested the naïve T cells before starting the time-series stimulation for all conditions and time points in parallel, and finally lysed the cells as fast and comparably as possible; all in a carefully balanced plate design to additionally avoid batch effects or influences of processing order. Other impactful studies have similarly measured the time-course gene expression on the population level during differentiation of other CD4 T cell subsets (Th1, Th2, Th17) where potential sorting markers are also expressed later than early genes of interest [60–63]. These studies successfully identified important genes, although the fraction of the desired differentiated cells were often lower than the fraction of FOXP3+ iTregs in our study. Most importantly, the genes we termed as DEGs in iTregs were defined in comparison to Mock-stimulated cells, and the latter also upregulated CD25 upon activation. Although there were protocol-specific differences in the absolute fraction of CD25+ cells, it was generally in a similar range in both iTregs (except those induced in the presence of Rapa) and Mock-stimulated cells; the latter expressed CD25 but not FOXP3 even when gated on CD25+ cells. Expression of each gene was modeled over time and the candidate DEGs and iTreg subnetwork genes were defined by comparing iTregs to Mock-stimulated cells in this time-series analysis. In particular, candidate gene expression was usually relatively low in naïve and Mock-stimulated cells, while being upregulated in iTregs under all conditions (including the Rapa-containing condition) and often already at early time points (before CD25 would be expressed). In line with the well-known proliferation-inhibiting effect of Rapa [29, 64], we observed by PCA that, on a global scale, Rapa-induced iTregs seemed to ‘lag behind in time’ and the time-course modeling of the genes would likely reveal iTreg-specific genes (vs. Mock-stimulated cells) even if the kinetics were different, which would not be possible with a simple sample-to-sample comparison at a single time point. Nevertheless, Rapa-induced iTregs displayed many unique genes compared to other iTregs, which may be partially attributable to the lower fraction of activated cells but also to specific Rapa-induced effects. Particularly for global comparisons of all activation-induced (not only iTreg specific) genes, it should be considered that Rapa-induced iTreg populations contained lower fractions of activated cells; however, this study is focused on iTreg-specific genes shared between different iTreg protocols (vs. Mock stimulation).

Despite these limitations, novel Treg regulatory molecules with differential expression were revealed here and original findings relevant for human immunology are discussed below, both from a global genomic perspective and a gene-centric view. For example, although the suppressive capacity of iTregs is controversial and strongly depends on the particular inducing and resting conditions, suppression assay setup, and controls used [27–29], our data may be useful to define suppressive cell markers and genes necessary for suppressive function, as we provide data from iTregs generated under several conditions and displaying varying degrees of suppressive activity, along with nTregs. Interestingly, and as discussed previously [28], iTregs induced with TGF-β + ATRA + Rapa displayed enhanced suppressive activity in vitro despite the lowest FOXP3 fraction compared to other iTreg populations, which may be related to the lower expression of ‘Treg down’ signature genes in TGF-β + ATRA + Rapa iTregs, modulation of TCR signal strength, or inhibition of contaminating ‘non-Treg’ proliferation by Rapa. Here, the higher sensitivity of conventional T cells compared to Tregs to inhibition by Rapa [65] may allow for a preferential expansion of Treg-like cells despite lower total fractions of activated cells in the presence of Rapa and may explain the relatively high expression of iTreg candidate genes despite the lower CD25+ cell fraction. Very recently, Candia et al. [66] re-sorted CD4^+^CD25^high^CD127^−^ cells from iTreg cultures prior to suppression assays and confirmed that human iTregs generated in the presence of Rapa had superior suppressive activity in vitro compared to the corresponding iTregs generated without Rapa. However, the in vivo relevance and functionality of iTregs is still controversial, and even iTregs generated with similar protocols are not always determined to be suppressive in vitro and/or in vivo. For instance, although we previously determined that iTregs were not suppressive in vivo in a xenogeneic GvHD model [28], others have shown that iTregs generated with similar protocols could suppress disease in vivo [67–69]. In addition to the technical details that may compromise comparability of such assays between different studies, it is also possible that different subsets of Tregs may suppress only certain target cell types and/or in specific tissues. Tregs employ numerous suppressive mechanisms [70], and in recent years it emerged that in vivo-generated nTregs are highly heterogeneous with tissue-specific phenotypic and functional specialization of nTregs beyond the classification of pTreg and tTreg subsets [40–42]. For instance, Tregs residing in the gut, which may mostly comprise pTregs and may resemble ATRA-induced iTregs, are different from Tregs in other tissues [16, 41]. In this regard, it is important to mention that ATRA and Rapa were shown to confer differential homing capacities to murine and human iTregs [66, 71]. It is therefore conceivable that iTregs with a certain phenotype may be suppressive in one, but not in another disease model. Importantly, although the in vivo relevance of iTregs generated in this study remains unclear, iTregs are the only applicable system to study FOXP3 induction in human primary cells. Further, we have confirmed the highly reproducible molecular patterns of iTregs on multiple levels (RNA, protein) and provided several controls alongside. The identification of several well-known Treg regulators in our study supports the relevance of our results for FOXP3 expression in human T cells.

On a global scale, our integration of data from primary human T cells revealed interesting aspects of the protein:RNA correlation during a major cellular transition like T cell activation and differentiation. About half of the molecules being both DEG and DEP showed a concordant pattern over time, while the other half presented either poor or negative correlation, in line with previous works reporting that RNA levels can only poorly predict protein abundance [44, 72, 73] and that gene-specific correction factors can improve the consistency between RNA levels and protein copy number [74]. For example, we observed a negative RNA:protein correlation among mitochondrial ‘OXPHOS’ genes, which has been previously observed in tumor cells [73]. We further identified a group of genes enriched in ribosomal and nucleolar functions with a poor RNA:protein correlation, which may be explained by previously reported mechanisms, such as low translational efficiency, as observed for members of the TOP mRNA family, which comprises components of the translational machinery, including ribosomal proteins and some small nucleolar RNAs [75]. Interestingly, both metabolism-associated and ribosomal proteins have been recently investigated as a signature to define the functional specialization of human nTregs [76]. Thus, despite the relatively high overlap between DEPs and DEGs, our integrative analysis underlines the importance of studying not only transcript but also protein abundance. While transcriptional data, due to its richness, coverage, and cost efficiency, may allow for more stringent statistical analyses and sample throughput, proteomics data are a valuable addition to narrow down findings from RNA data in order to obtain more confidence for defining differentially expressed molecules and selecting those for perturbation and functional validation approaches.

If a gene set-focused level is required, based on our study, we propose novel iTreg regulators, the function of which was validated by demonstrating that knocking them down in an shRNA screen prevented FOXP3 expression. It should be noted that decreased FOXP3 expression upon candidate knockdown may not be related solely to altered FOXP3 induction, and that a role of candidate molecules in FOXP3 maintenance is also possible. Yet, the expression kinetics of most candidate molecules being expressed prior to FOXP3, as well as the functional assignment of many candidate molecules to the TGF-β pathway cluster together with the known role of the TGF-β response element in FOXP3 induction [77], suggest a role in FOXP3 induction at least for the respective candidate molecules. Further, except for one candidate gene (*OLFM2*), ectopic FOXP3 expression in human T cells in an external dataset did not cause differential expression. Particularly for those candidate molecules that are TFs, a direct regulation of the FOXP3 gene is a possibility, but FOXP3 expression as a readout cannot distinguish between direct and indirect regulation.

Future work has to be performed to confirm and extend the findings from the shRNA validation screen and the gene expression studies, such as assessing the molecular mechanisms through which candidate molecules affect FOXP3 expression, exploration of the candidate molecules in other cell types including CD4+ T cell subsets, and studying the relevance of the molecules in Tregs in vivo*.* Furthermore, despite protocol optimization and careful design of the shRNA screen, including several negative and positive controls, we cannot completely exclude off-target effects or divergent knockdown efficiencies of individual shRNA clones. This should be addressed in the future by deconvoluting individual shRNA effects, correlating knockdown efficiency with the effect size on FOXP3, and confirming the results for each candidate molecule in detail with independent assays.

Despite the limitations of this study, it is tempting to speculate on the potential of the candidate genes as novel drug targets. They belong to several molecular classes amenable to different drug targeting approaches, as they include TFs, surface molecules, and members of the TGF-β signaling pathway. For example, the candidate TF NR3C1 (glucocorticoid receptor) is already a well-known drug target [78], and its effects on iTregs should be specifically investigated further in the future. Interestingly, the TF TRIM22 selected by integrating iTreg RNA and protein data and having strong effects on FOXP3 in the validation screen does not have a murine homologue and was recently described to be associated with early-onset IBD [79], emphasizing the importance of studying human instead of murine T cells.

The data presented here may be useful in future investigations to discover new markers to define Treg subsets. Indeed, our new candidate molecules outperformed known Treg regulators in classifying iTregs versus Mock-stimulated T cells. Although the LDA approach is subject to the selection bias [53], we confirmed a good performance of the candidate molecules in the independent dataset, and we also found the candidate molecules strongly represented in the top-ranking 100 and even top 20 genes obtained from unbiased RF analysis, further supporting our results at least for a subset of candidate molecules. In addition to discrimination of iTregs from Mock-stimulated cells, the classifiers could potentially be useful for pTreg versus tTreg discrimination as well, which would be relevant for specific targeting of either subset depending on the disease. Due to similar induction requirements (IL-2 and TGF-β) it may be speculated that iTregs are likely to be more similar to pTregs than to tTregs. Furthermore, pTregs and tTregs differ in their TCR repertoire [1, 80] and, in this respect, due to the common naïve T cell precursor, iTregs might be similar to pTregs; nevertheless, in vivo, only a small fraction of pTreg and conventional T cell TCR sequences seem to overlap [1]. Unfortunately, in contrast to murine cells where Neuropilin-1 and Helios can distinguish pTregs and tTregs at least under most non-inflammatory conditions, these markers are not usable for such distinction in human cells [1]. In accordance with our data, murine nTregs and iTregs were shown to differ in parts of their signature while, experimentally, in vivo-induced murine pTregs were more similar to nTregs [3, 38, 39], arguing for additional factors during in vivo Treg induction. Yet, signatures of in vivo-induced pTregs differ depending on the mode of conversion and, regarding the Treg signature genes, pTregs still considerably differ from ex vivo-isolated (splenic or lymph node) Tregs while much of the in vivo-converted Treg signature overlaps with in vitro-converted iTregs [39]; these observations may reflect imprinting of tissue-specific signatures. Furthermore, the activation with anti-CD3/-CD28 largely shapes the gene signature of in vitro differentiating cells as evident from our time-series data, and is therefore likely to at least partially contribute to the observed difference between iTregs and ex vivo-derived (unstimulated) nTregs. Cell number limitations did not allow us to include activated nTregs in this study, but in accordance with this notion, a drastic change of the human nTreg gene signature upon in vitro activation has been described along with a shared activation signature between activated Tregs and activated Tcons [81, 82]. It should also be noted, again in this context, that nTregs comprise different subsets [40, 42] and can be isolated to different grades of purity; and, in our study, nTregs were isolated solely on the basis of high CD25 expression because of the relatively high cell numbers required for proteomics. These bulk ‘nTregs’ supposedly include pTreg and tTreg subsets; the latter may be prominent according to studies in mice from which it has been estimated that more than 70% of Tregs in the periphery are tTregs [1]. A similar proportion has been proposed for human Tregs in peripheral blood, although use of the marker Helios has been debated [1]. Thus, differences between the nTreg and iTreg signatures observed in the present study might reflect true differences in iTregs versus nTregs but also pTregs versus tTregs. Despite the controversies regarding Helios as a tTreg marker [1], it is worth mentioning that, in line with the notion of iTregs being more similar to pTregs, iTregs induced under all conditions and in both datasets of the present work displayed low expression of *IKZF2* (encoding for Helios) not exceeding that of Mock-stimulated or unstimulated cells, while nTregs expressed drastically higher levels of *IKZF2* mRNA and Helios protein (data not displayed). Interestingly, iTregs in this study expressed *IKZF4* (Eos) but not *IKZF2* (Helios), both of which were previously shown to be expressed in a FOXP3-independent manner and suggested to correlate with an nTreg-specific DNA methylation signature [26, 57]. However, the Treg epigenetic signature, such as DNA demethylation of the Treg-specific demethylated region in the *FOXP3* gene, which is important for stable FOXP3 expression, appears divergent in iTregs versus nTregs (including in in vivo-induced pTregs in some studies) [2, 25, 26, 28, 57]. Yet, relative instability of pTregs may even be a favorable scenario in vivo, conferring local and transient immune suppression in specific tissues, in contrast to long-lasting, stable tolerance to self-antigens mediated by tTregs and preventing autoimmune disease. Interestingly, deletion of the element containing the Treg-specific demethylated region in the FOXP3 locus did not affect either FOXP3+ thymocyte numbers in vivo or FOXP3 induction, but compromised Treg stability, with functional relevance in chronic infectious settings or in older mice [83–85]. Irrespective of the differences between nTregs and iTregs, the latter appear as the most suitable system to study early events of FOXP3 induction in human cells. Indeed, our approach using differentiation of iTregs enabled the identification of several well-known nTreg factors, along with novel molecules for which we confirmed a functional role in in vitro FOXP3 induction.

## Conclusion

In conclusion, we provide a valuable data resource with RNA-Seq and corresponding proteomics time-series data of primary human T cells, comprising naïve T cells, activated T cells, iTregs induced by four different protocols, and nTregs. These data can be exploited in the future for detailed analyses of iTreg signatures, including specific effects of Treg-inducing compounds and discovery of markers to distinguish Treg subsets. We present a set of 37 novel candidate molecules, of which several functionally affected induction of FOXP3+ iTreg cells in a shRNA screening setting and could classify iTregs versus Mock-stimulated T cells. These candidate molecules have the potential to be developed further for use as drug targets in autoimmune and inflammatory diseases or cancer, given the known relevance of FOXP3+ Tregs in these diseases.

## Methods

### Human T cell isolations

Anonymized healthy donor buffy coats were purchased from the Karolinska University Hospital (Karolinska Universitetssjukhuset), Huddinge, Sweden. The study was approved by the Regional Ethical Review Board in Stockholm (Regionala etikprövningsnämnden i Stockholm), Sweden (approval number: 2013/1458–31/1). Fresh buffy coats were filled with PBS to 170 mL and used to isolate human peripheral blood mononuclear cells (PBMCs) by gradient centrifugation using Ficoll-Paque Plus (GE Healthcare), followed by monocyte depletion through plastic adherence in RPMI medium containing 10% FCS (Invitrogen). Afterwards, magnetic activated cell sorting (MACS) was utilized to isolate T cell subsets from PBMCs: CD25^high^ ‘nTregs’ were first prepared by positive isolation as described previously [86] using limited amounts (2 μL/10^7^ cells) of CD25 beads (Miltenyi) and two subsequent MACS columns. Platelets were removed from PBMCs by low-speed centrifugation (200× *g*, 5–10 min, 20 °C; 3–4 times), followed by isolation of naïve CD4+ T cells from the nTreg-depleted fraction by negative isolation using the naïve CD4+ T Cell Isolation Kit II, human (Miltenyi), according to the manufacturer’s instructions. In brief, CD45RO+ memory T cells and non-CD4+ T cells were indirectly magnetically labeled using a cocktail of biotin-conjugated antibodies against CD8, CD14, CD15, CD16, CD19, CD25, CD34, CD36, CD45RO, CD56, CD123, TCRγ/δ, HLA-DR, and CD235a. For shRNA validation screening, fresh blood was obtained from anonymized healthy donors after informed consent at the La Jolla Institute for Allergy and Immunology, La Jolla, USA, according to institutional guidelines (Normal Blood Donor Program protocol VD-057). Blood was diluted 1:2 with PBS and PBMCs and naïve CD4+ T cells were isolated as above. The purity of MACS-isolated cells was assessed by flow cytometry. Cells were counted in trypan blue solution using a Countess Automated Cell Counter (Life Technologies) and viability was determined by trypan blue staining and/or flow cytometry (see below). T cells were cultured at 5% CO_2_/37 °C.

### iTreg differentiation for molecular profiling

Naïve CD4+ T cells were isolated from buffy coats of male anonymous donors (aged 37, 38, and 34 years) by MACS as described above, rested for several hours and then plated under iTreg differentiation conditions as described previously [28, 29]. Briefly, cells were stimulated with 5 μg/mL plate-bound anti-CD3 antibody (clone OKT3; Biolegend, LEAF grade), 1 μg/mL soluble anti-CD28 antibody (clone CD28.2; Biolegend, LEAF grade), and 100 IU/mL IL-2 (carrier-free; R&D Systems). ‘Mock’ control cells received no further compounds. For Treg-inducing conditions, TGF-β1 (5 ng/mL carrier-free; R&D Systems), ATRA (10 nM; Sigma-Aldrich), Rapa (100 ng/mL; Calbiochem EMD Millipore), or sodium butyrate (100 μM; Sigma-Aldrich) were added. Plates were prepared first, pre-warmed to 37 °C, and cells added last. Cells were plated in 96 U-well plates at 1.0–1.2 × 10^5^ cells/well, in a 200 μL final volume/well serum-free X-Vivo 15 medium (Lonza) supplemented with Glutamax (Gibco). Margin wells were not used for cell culture, and all unused wells were filled with 200 μL PBS. The plating layout was balanced for iTreg conditions and time points within and across donors to prevent any batch effects through processing order or culturing samples on the same plate. Samples for each donor were prepared in completely independent experiments but with reagents aliquoted from the same batches. Cells were incubated for 2 h to 6 days. At the indicated time points (2, 6, 24, and 48 h, and 6 days), several wells per condition were pooled (between 5 and 30 wells, considering cell growth for conditions and time points) and cells were processed for RNA and protein extraction. On days 4 and 6, 1–2 wells of each iTreg condition were used for flow cytometric analysis (see below).

### RNA and protein extraction for molecular profiling

At the indicated time points, the plates were placed on ice, 100 μL of the supernatant were removed, and 100 μL of ice-cold X-Vivo 15 medium was added to each well. Cells were processed in the order of plating according to the experimental design, balanced for donor and condition to prevent batch effects by processing time. Replicate wells were pooled with a multichannel pipet, rinsed with cold PBS, and the cells transferred to 15 mL tubes. Cells were centrifuged (450× *g*, 10 min, 4 °C), the supernatant removed, and the cells were washed twice with 1 mL of ice-cold PBS each (1000× *g*, 5 min, 4 °C). The supernatant was removed completely, cells were vortexed and lysed in RLT buffer (Qiagen) supplemented with 142 mM beta-mercaptoethanol (Sigma Aldrich), and homogenized using Qia Shredder columns (Qiagen). Lysates were frozen on dry ice and stored at −80 °C until processing. RNA and proteins were extracted with the AllPrep DNA/RNA/Protein Mini Kit (Qiagen) according to the manufacturer’s recommendations with the following modifications. Centrifugations were carried out at 9600× *g* except for protein precipitation, which was at 20,800× *g*. RNA was eluted with 40 μL of RNase-free water and elution was repeated with the first eluate. An aliquot of RNA was taken for nanodrop, bioanalyzer, and qRT-PCR, and the remaining RNA was frozen on dry ice and stored at −80 °C. The protein precipitate was solubilized (5 min, 95 °C) in freshly prepared buffer containing 4% (w/v) SDS, 25 mM HEPES pH 7.6, and 1 mM DTT, and the insoluble material was removed by centrifuging for 1 min at 20,800× *g*. An aliquot of the soluble protein supernatant was taken for BCA assay, and the extracts were frozen on dry ice and stored at −80 °C. The BCA assay of 1:5 diluted samples was performed according to the manufacturer (Pierce BCA Protein Assay Kit, Thermo Fisher Scientific) in a 96-well format; an aliquot of the lysis buffer (frozen along with the sample aliquots) was used (1:5 diluted) to prepare the standard curve with BSA protein. All samples were used for RNA-Seq. All samples except the 2 h time point samples, iTreg TGF-β + ATRA and iTreg TGF-β + butyrate, were used for proteomics.

### RNA sequencing (RNA-Seq)

RNA concentration was measured on a Nanodrop 2000 spectrophotometer (Thermo Fisher Scientific). RNA quality was controlled using an Agilent RNA 6000 Pico Kit on a 2100 Bioanalyzer instrument (Agilent Technologies). RNA integrity numbers were 8.47 ± 0.50 (mean ± SD; range 7.6–10.0); 1 μg of RNA per sample (except for the 2 h time point samples of Donor 2: 500 to 900 ng) was used for library preparation with the TruSeq Stranded mRNA HT kit (Illumina), with Dual Indexing adapters (8-plex). As a control, an Ambion ERCC Spike-In Control Mix was used (Thermo Fisher Scientific). According to the standard protocol, libraries were purified from excess sequencing adapters with Agencourt AMPure XP beads (Beckman Coulter). Libraries were generated in two batches, in which samples were balanced across a library batch and adapter 8-plex according to donor, iTreg/control condition, and time point to allow for the accounting of potential batch effects. Library concentration was measured on a Qubit 2.0 Fluorometer (Thermo Fisher Scientific) and library quality and size were determined by using an Agilent High Sensitivity DNA Kit on a 2100 Bioanalyzer instrument (Agilent Technologies). Libraries were quantified with the KAPA Library Quantification Kit (KAPA Biosystems). Pools of eight libraries (except for one lane, nine libraries) were denatured using NaOH, and PhiX Control v3 was added to each pool. Pools were clustered and sequenced in two batches, using one High Output Run and one Rapid Run, on a HiSeq 2500 Sequencing Platform (Illumina). For the High Output run, library pools were clustered on a HiSeq Flow Cell v3 on the cBot system, using TruSeq PE Cluster Kit v3 - cBot - HS, and Read 1 Sequencing Primer Mix (HP10) from a TruSeq Dual Index Sequencing Primer Kit. Clustered libraries were then sequenced with 75 nt paired-end reads, using TruSeq SBS Kit v3 50 cycle kits. The Index 1 sequencing primer (HP12) and Read 2 sequencing primer (HP11) provided in the TruSeq Dual Index Sequencing Primer Kit were used. For the Rapid run, library pools were clustered on a HiSeq Rapid Flow Cell v1 using TruSeq Rapid Duo cBot Sample Loading Kit. Clustered libraries were then sequenced using a TruSeq Rapid PE Cluster Kit together with three TruSeq Rapid SBS 50 cycle kits. On average, 18.9 million read pairs were obtained per sample (mean ± SD 18.9 × 10^6^ ± 5.23 × 10^6^; range 5.3 × 10^6^ to 32.1 × 10^6^). BCL basecall files were de-multiplexed and converted to FASTQ files with bcl2fastq v1.8.4.

### Quantitative reverse transcriptase PCR (qRT-PCR)

An aliquot of RNA used for sequencing, and from an additional donor, was taken for qRT-PCR analysis. cDNA was prepared with the SuperScript VILO cDNA Synthesis Kit (Invitrogen) according to the manufacturer’s instructions. mRNA was quantified with gene expression mastermixes for SYBR Green or Taqman method (Applied Biosystems) and measured on a StepOne plus detector system (Applied Biosystems) as follows. *IKZF4* and *RPL13A* mRNA was measured using Applied Biosystems best coverage Taqman probes (FAM reporter), and *FOXP3* and *RPL13A* mRNA was measured by SYBR Green qRT-PCR (primers: *FOXP3* Forward: AGCTGGAGTTCCGCAAGAAAC, *FOXP3* Reverse: TGTTCGTCCATCCTCCTTTCC; *RPL13A* Forward: TCCAAGCGGCTGCCGAAGATG, *RPL13A* Reverse: CTTCCGGCCCAGCAGTACCTGT). The relative mRNA expression was determined by normalization to *RPL13A* and results are presented as fold induction compared to mRNA amounts of unstimulated T naïve of the same donor, which were set to 1. Fold expression was calculated using the ∆∆Ct method according to the following formula (Ct is the threshold cycle value):

$$ \mathrm{Relative}\ \mathrm{mRNA}\ \mathrm{expression}={2}^{\hbox{--} \left( Ct\  of\ gene\ of\ interest\hbox{--} Ct\  of\  RPL 1 3A\right)} $$

### Proteomics

#### Sample preparation for mass spectrometry

At least 25 μg of protein per sample from Allprep extraction (see above; in 4% SDS, 25 mM HEPES, 1 mM DTT) was used for proteomics. Lysates were heated to 95 °C for 5 min followed by sonication for 1 min and centrifugation at 14,000× *g* for 15 min. The supernatant was mixed with 1 mM DTT, 8 M urea, and 25 mM HEPES at pH 7.6, and transferred to a 10-kDa cut-off centrifugation filtering unit (Pall, Nanosep), and centrifuged at 14,000× *g* for 15 min. Proteins were alkylated by 50 mM iodoacetamide in 8 M urea and 25 mM HEPES for 10 min. The proteins were then centrifuged at 14,000× *g* for 15 min followed by two more additions and centrifugations with 8 M urea and 25 mM HEPES. Trypsin (Promega) in 250 mM urea and 50 mM HEPES was added to the cell lysate at a ratio of 1:50 trypsin:protein and incubated overnight at 37 °C with gentle shaking. The filter units were centrifuged at 14,000× *g* for 15 min followed by another centrifugation with ultra-pure water (Milli-Q, Millipore) and the flow-through was collected. Peptides were labelled with TMT10-plex reagent according to the manufacturer’s protocol (Thermo Fisher Scientific) and cleaned by a strata-X-C-cartridge (Phenomenex). Samples for the same culture condition within a donor were kept together in a 10-plex. A slight batch effect dependent on the TMT set (Additional file 1: Figure S3) may be explained by the distribution of individual donors within each TMT set, which was performed to increase quantification overlap. In total, five 10-plexes were used, and an internal standard containing a mix of protein from all samples was used as one TMT tag in each TMT set to enable relative quantification between TMT sets. Some samples were loaded in duplicate in different 10-plexes.

#### Immobilized pH gradient – isoelectric focusing (IPG-IEF) of peptides

The TMT-labeled peptides, 250 μg per TMT-10-plex, were separated by IPG-IEF on a 3–10 strip*.* Peptides were extracted from the strips into 72 fractions by a prototype liquid handling robot, supplied by GE Healthcare Bio-Sciences AB. A plastic device with 72 wells was put onto each strip and 50 μL of ultra-pure water (Milli-Q, Millipore) was added to each well. After 30 min incubation, the liquid was transferred to a 96-well plate and the extraction was repeated two more times. The extracted peptides were dried in a SpeedVac vacuum concentrator and dissolved in 3% acetonitrile and 0.1% formic acid.

#### Q Exactive analysis

Before analysis on the Q Exactive Hybrid Quadrupole-Orbitrap Mass Spectrometer (Thermo Fischer Scientific), peptides were separated using an Ultimate 3000 RSLCnano system. Samples were trapped on an Acclaim PepMap nanotrap column (C18, 3 μm, 100 Å, 75 μm × 20 mm), and separated on an Acclaim PepMap RSLC column (C18, 2 μm, 100 Å, 75 μm × 50 cm; Thermo Fisher Scientific). Peptides were separated using a gradient of A (5% DMSO, 0.1% formic acid) and B (90% acetonitrile, 5% DMSO, 0.1% formic acid), ranging from 6% to 37% B in 30–90 min (depending on IPG-IEF fraction complexity) with a flow of 0.25 μL/min. The Q Exactive was operated in a data-dependent manner, selecting the top 10 precursors for fragmentation by high collision dissociation (HCD). The survey scan was performed at 70,000 resolution from 400 to 1600 m/z, with a max injection time of 100 ms and target of 1 × 10^6^ ions. For generation of HCD fragmentation spectra, a max ion injection time of 140 ms and automatic gain control of 1 × 10^5^ were used before fragmentation at 30% normalized collision energy and 35,000 resolution. Precursors were isolated with a width of 2 m/z and put on the exclusion list for 70 s. Single and unassigned charge states were rejected from precursor selection.

#### Peptide and protein identification

All Orbitrap data was searched by SequestHT under the software platform Proteome Discoverer 1.4 (Thermo Fisher Scientific) against the Ensembl 81 human protein database and filtered to a 1% FDR. A precursor mass tolerance of 10 ppm and product mass tolerances of 0.02 Da for HCD-Fourier Transform Mass Spectrometry (FTMS) were used. Further settings used were trypsin with two missed cleavage; iodoacetamide on cysteine and TMT on lysine and N-terminal as fixed modifications; and oxidation of methionine as variable modification. Quantification of TMT-10-plex reporter ions was performed by Proteome Discoverer on HCD-FTMS tandem mass spectra using an integration window tolerance of 10 ppm. Only peptides unique to a protein group were used for quantitation.

### Western blot

T cells were cultured and harvested as described above, and protein extraction was performed as above for molecular profiling. Protein concentrations were determined by BCA assay and 25 μg of protein per lane were resolved by SDS-PAGE using Mini-PROTEAN TGX Gel (Bio-Rad). Proteins were transferred by standard Wetblot procedures to Protran nitrocellulose membranes (Amersham GE Healthcare). Membranes were blocked with 5% nonfat dry milk in TBS containing 0.1% (w/v) Tween 20. The primary antibodies used were the following: anti-FOXP3 (clone eBio7979, eBioscience), anti-PARP (clone C2–10, BD Biosciences), anti-GAPDH (clone 6C5, Santa Cruz Biotechnology), anti-alpha-Tubulin (clone B-5-1-2, Sigma-Aldrich), and anti-STIM1 (N-terminal, rabbit polyclonal, Sigma-Aldrich). The FOXP3 antibody clone eBio7979 recognizes an epitope between exon 3 and the Zink finger domain, and should recognize all three known FOXP3 isoforms. Several proteins were used as loading controls, since ‘housekeeping’ proteins GAPDH and alpha-tubulin were differentially expressed between samples. Horseradish peroxidase-conjugated secondary antibodies were from Santa Cruz Biotechnology, and protein bands were developed in a Vilber Fusion Solo S chemiluminescence acquisition system (Vilber Lourmat) using Immobilon Western Chemiluminescent horseradish peroxidase substrate (Millipore). Only non-saturated exposures were used for visualization and analysis, and bands were quantified with ImageJ software version 1.48v.

### Flow cytometry and antibodies

An aliquot of cells used for molecular profiling was stained for flow cytometry on days 4 and 6 of culture. Day 4 samples were stored after fixation and processed further together with day 6 samples. On day 6, samples were stained without and after 4 h restimulation with 0.5 μM ionomycin and 10 ng/mL of phorbol 12-myristate 13-acetate (Sigma Aldrich) in the presence of Golgi plug (BD Biosciences). If not otherwise stated, staining was performed as follows:

- Cell surface antigen staining with the following antibodies: CD4-PerCP (clone SK3, BD Biosciences), CD45RA-PE-Vio770 (clone T6D11), CD45RA-FITC (clone T6D11), and CD25-PE (clone 4E3; all Miltenyi Biotec) were performed in the dark with antibody dilutions in FACS buffer (PBS/0.5% HSA) for 30 min at 4 °C. Cells were washed once with PBS, resuspended in FACS buffer, and acquired or used for subsequent intracellular staining. After surface staining, cells were washed twice with PBS and stained with the Fixable Viability Dye eFlour780 (ebioscience) for 30 min at 4 °C (dark), then washed twice and used for intracellular staining.
- Intracellular staining was performed at 4 °C with the Foxp3 Staining Buffer Set (ebioscience) according to the manufacturer’s protocol using the following antibodies: FOXP3-APC (ebioscience, clone 236A/E7), CTLA-4-BV421 (BD Biosciences, clone BNI3), and IFN-γ-FITC (ebioscience, clone 4S.B3). Isotype control antibodies (mIgG1κ APC isotype, ebioscience, clone P3.6.2.8.1; mIgG2aκ BV421 isotype, BD Biosciences, clone MOPC-173; mIgG1κ FITC isotype, ebioscience, clone: P3.6.2.8.1) were used at the same final concentrations (w/v) as the corresponding intracellular staining antibodies.

#### Acquisition and analysis

If not otherwise stated, acquisition was performed on a CyAn ADP 9 Color Analyzer (Beckman Coulter), and parameter compensation was performed automatically with the CyAn software (Summit) tool using single stained samples containing positive cells. Flow cytometry raw data were analyzed using FlowJo Software (Tree Star) and exported values for cell fractions were analyzed in GraphPad Prism 6 or R.

### Lentiviral transduction of T cells for shRNA validation screen

shRNA libraries used were based on the pLKO.1-puro TRC vector backbone developed by The RNAi Consortium (TRC). TRC1, TRC1.5, or TRC2 clones from the Sigma Mission TRC - Human collection (Sigma Aldrich) were used, cherry-picked, and obtained as bacterial glycerol stocks from the RNAi center at the La Jolla Institute for Allergy and Immunology (for clone list, see Additional file 8: Table S7). Bacteria were inoculated in overnight cultures and pLKO.1 plasmids were extracted individually with the E.Z.N.A. Plasmid DNA Mini Kit I (Omega Bio-tek) according to the manufacturer’s instructions with an optional second washing step and eluted in sterile 10 mM Tris at pH 8.0. Empty pLKO.1 vector or pLKO.1-Scr (with scrambled non-targeting shRNA) were used as negative controls, and pLKO3G (encoding for GFP in place of puromycin resistance gene) was used to control for transfection and transduction efficiency. A pool of clones targeting human CD4 was used to optimize T cell transduction and knockdown. For knockdown of ‘iTreg candidate genes’ , a pool of 2 to 8 (mean 4.3, SD 0.9) shRNA-encoding plasmids per gene was transfected to 293T cells in equal amounts to produce lentivirus. For some genes, two independent pools of shRNA-encoding plasmids were used. Each pool contained only shRNA-encoding plasmids from the same TRC version (for list of clones, see Additional file 8: Table S7).

#### Lentivirus production

293T cells (derivative of human embryonic kidney 293 cells containing the SV40 T-antigen) were obtained from ATCC and tested negative for mycoplasma. 293T cells were harvested at 60–85% confluency and seeded at 4 × 10^5^ cells per well in six-well plates in 2 mL of antibiotic-free DMEM high-glucose medium supplemented with 10% FBS and 2 mM glutamate (Gibco). After 20–23 h (confluency 40–60%), the medium was replaced with 2 mL of fresh pre-warmed medium and 1–2 h later, 293T cells were transfected for lentivirus production. Cells were co-transfected with pLKO.1 shRNA transfer vectors (see above) along with packaging and pseudotyping vectors (pCMV-dR8.9 and pCMV-VSV-G, a kind gift from Sara Trifari, La Jolla Institute for Allergy and Immunology) using jetPRIME transfection reagent according to the manufacturer’s recommendations (Polyplus transfection). After 4 h, the medium was removed and replaced by 1 mL of fresh pre-warmed medium. Transfection efficiency was estimated after approximately 24 h in samples transfected with pLKO3G to be above 95% by fluorescence microscopy. The virus-containing supernatant was harvested 38 h after transfection and centrifuged at 350× *g* for 5 min to remove cellular material, and used on the same day to transduce T cells.

#### T cell transduction and culture

Human naïve CD4+ T cells were pre-activated for 16–18 h with plate-bound anti-CD3 antibody (coated at 5 μg/mL), soluble anti-CD28 antibody (1 μg/mL), and 100 U/mL of IL-2 in 96 U-well plates at 130,000 cells/well in 200 μL of X-Vivo 15 medium. Margin wells of 96 U-well plates were not used for culturing but instead filled with 200 μL of PBS. T cell plates were centrifuged (450× *g*, 10 min) and 165 μL of medium was removed and kept in the incubator as T cell-conditioned medium. T cells were transduced with 200 μL/well of fresh viral supernatant with a final concentration of 8 μg/mL of Polybrene (hexadimethrine bromide, Millipore) by spin infection (900× *g*, 1 h, 35 °C). T cells were subsequently cultured for 3 h at 37 °C/5% CO_2_, then centrifuged (500× *g*, 5 min), and viral supernatants were removed and replaced by T cell-conditioned medium. At 26 h after transduction, T cell differentiation was started. Differentiation plates coated with anti-CD3 antibody were prepared with compounds for iTreg conditions (anti-CD28 + IL-2 + TGF-β1 + ATRA) or ‘Mock’ stimulation (anti-CD28 + IL-2) as described above, with additional supplementation of medium with 1 μg/mL of puromycin (Gibco) except for controls without the puromycin resistance gene. Transduced T cells and controls were centrifuged (500× *g*, 10 min), the medium was removed, and cells were washed twice with 200 μL of pre-warmed X-Vivo 15 medium. T cells were resuspended in 60 μL of X-Vivo 15 medium, and 50 μL of the cell suspension were transferred to pre-warmed prepared differentiation plates (one well each for iTreg and Mock stimulation per targeted gene and donor). The lack of hindrance of FOXP3 induction by pre-activation, transduction, or puromycin selection had been previously tested. After 4–5 days of differentiation culture, T cells were stained for viability and expression of CD25, CD45RA, and FOXP3, as described above, except for control cells transduced with pLKO3G, which were only stained for viability and then fixed with 2% paraformaldehyde (methanol-free) in PBS instead of the FOXP3 buffer set. Samples were acquired on a FACS Canto II cytometer (BD Biosciences) and compensation was performed with the in-build compensation tool (BD FACSDiva software) using single stained samples.

### RNA-Seq data analysis

FASTQ files were adapter- and quality-trimmed using Trim Galore!, and aligned with TopHat2 using hg19 genome index and GENCODE v19 transcriptome model. Alignment metrics and statistics were extracted from the BAM files using Picard tools. Reads were summarized to the GENCODE v19 genes with the summarizeOverlaps function of the GenomicAlignments [87] package. Unmapped reads were mapped with bowtie2 against an ERCC index in order to obtain ERCC spike-in counts and FPKM values and to explore the dynamic range.

Exploratory analysis and visualization (Additional file 1: Figure S3) was performed using DESeq2 R package [88], after removing the genes with zero counts in all samples and normalization using the DESeq2 size factors. Blind regularized logarithmic transformation (rlog) was applied to the count matrix in order to calculate Euclidean distances and correlations between samples. PCA on rlog-transformed data and multidimensional scaling using Poisson distance on counts were also used to visualize sample-to-sample relationships. To explore the relative effect of technical and biological factors, a separate linear model was fitted between each of the PC scores and a series of explanatory variables and the R^2^ was calculated for each of them, obtaining a matrix of R^2^ values reflecting the association between the PCs and the explanatory variables.

#### Classification into LEGs and HEGs

We firstly obtained a threshold to define if a gene was considered expressed or not in the full dataset. It has been previously shown [36] that RNA-Seq can classify the metazoan genes, on the basis of the relative abundance of their mRNAs, into one group of lowly expressed and putatively non-functional genes (LEGs) and another group of HEGs (Additional file 1: Figure S6b). After verifying that the distribution of protein-coding genes in our dataset appears bimodal when reported as log_2_(FPKM) (fragments per kilobase per million mapped reads) values, we aggregated the log_2_(FPKM) values per group (G02–G06) and fitted a one-dimensional normal mixture model with two components and variable variance with mclust [89] to each group separately. After obtaining classification vectors, the midpoint between the maximum log_2_(FPKM) value of the LEGs and the minimum log_2_(FPKM) value of the HEGs was considered. A threshold (*t* = 1.496) was chosen as the average of all midpoints. Then, we considered a gene expressed in our samples if log_2_(FPKM) > t for at least 15 samples, obtaining 15,910 HEGs. RNA expression levels for candidate genes were further classified based on the range of expression considering all samples as follows: + < 100 counts; ++ ~100–1000 counts; +++ ~1000–10,000 counts (Additional file 1: Figure S7a).

#### Differential expression analysis

To model the differential expression over time, we used three methods based on R packages: (1) a DESeq2 [88] generalized linear model of the negative binomial family with time as a discrete factor; (2) a DESeq2 generalized linear model of the negative binomial family with a natural cubic spline of time; and (3) a maSigPro [90] third degree polynomial regression. We define the variable describing the different treatments as ‘group’, with the following levels: G01, unstimulated cells; G02, Mock-stimulated cells (control); G03, iTreg TGF-β; G04, iTreg TGF-β + ATRA; G05, iTreg TGF-β + ATRA + Rapa; G06, iTreg TGF-β + butyrate; and G07, unstimulated nTreg. Similarly, we define the following naming conventions for the variable ‘time point’: T01, 0 h; T02, 2 h; T03, 6 h; T04, 24 h; T05, 48 h; T06, 6 days.

For method (1), the samples for G01 and G07 were not included and we considered a design formula that models the group differences at time point 2 h (T02), the difference over time points, and any group-specific differences over time points (the interaction term) for each gene i and sample j:

$$ \log {\mu}_{ij}={\beta}_i^0+{\beta}_i^G{x}_j^G+{\beta}_i^T{x}_j^T+{\beta}_i^{GT}{x}_j^G{x}_j^T+\epsilon $$

With DESeq2, the dispersion was estimated and the model was fitted, and then a Wald test was performed for individual coefficients to extract log2(Fold Change) values, *p* values, and FDRs. Results were considered significant if FDR < 0.01. A likelihood ratio test was also performed comparing the full model with a model without the interaction term. For method (2), we operated similarly to method (1), but with a change in the design formula in order to model the gene expression as a smooth function of time, namely a natural cubic spline. The group difference and the interaction with the smooth function were also considered. Results were considered significant if FDR < 0.01. For method (3), we used the rlog-transformed data to perform a stepwise cubic regression and extract the best model for each gene using maSigPro, with the time and group as explanatory variables. Results were considered significant if FDR < 0.01 and R^2^ > 0.7.

To summarize the results, we assigned individual scores (G) counting the methods that defined a gene as being significantly differentially expressed. A gene was called a DEG if it was amongst the significant results of at least two of the three methods, corresponding to a score ≥ 2. In particular, we defined five different individual scores, corresponding to the time and the four different iTreg conditions (*G*_*time*_, *G*_*G03*_, *G*_*G04*_, *G*_*G05*_, *G*_*G06*_), plus a binary total score (*G*_*Sum*_), annotating if the gene was overall considered a DEG for at least one individual score. For the time comparisons, the following requirements were considered: for the DESeq2 methods (1) and (2), FDR < 0.01 for any base coefficient related to time; for the maSigPro method (3), FDR < 0.01 and R^2^ > 0.7 for the ‘control’ time series. For the group comparisons, we considered the following requirements: for methods (1) and (2), FDR < 0.01 for any coefficient of the ‘coefficient category’, i.e., the coefficient for the base variable ‘group’ and the all the corresponding interaction coefficients (‘group’ × ‘time’); for the maSigPro method (3), FDR < 0.01 and R^2^ > 0.7 for the time series corresponding to the ‘group’ variables.

### Proteomics data analysis

TMT ratio sample median normalization (ratio R vs. internal standard) was performed, using the median PSM TMT reporter ratio from peptides unique to a gene symbol. The protein relative quantification values (log_2_R) were used for exploratory analysis and visualization (Additional file 1: Figure S3), only considering the proteins detected in all samples. To explore sample-to-sample relationships, we used correlations between samples and PCA. To investigate the relative effect of technical and biological variables, a separate linear model was fitted between each of the PC scores and a series of explanatory variables and the R^2^ for each of them was calculated, obtaining a matrix of R^2^ values that was tested for associations between the PCs and the explanatory variables. To model the differential protein expression over time, samples for G01 and G07 were excluded and we used a design in limma R package [91] that models the group differences at baseline, the difference over time points, and the group-specific differences over time points (the interaction term), similarly to the method (1) for the detection of DEGs, but restricted to the time points and groups included in the proteomic experiments. Empirical Bayes statistics, *p* values, and FDR results were considered significant if FDR < 0.05.

To summarize the results, we created individual binary scores (0/1) to define DEPs. In particular, we defined three different individual scores, *P*, corresponding to the time and the two different iTreg conditions (*P*_*time*_, *P*_*G03*_, *P*_*G05*_), plus a binary total score (*P*_*Sum*_), annotating whether the protein was overall considered a DEP for at least one individual score. For the time comparisons, we considered FDR < 0.05 for any base coefficient related to time as a requirement; for the group comparisons, we required FDR < 0.05 for any coefficient of the ‘coefficient category’, i.e., the coefficient for the base variable ‘group’ and all the corresponding interaction coefficients (‘group’ × ‘time’).

The number of proteins per cell was estimated (for an average of all samples in which the respective protein was detected) according to Wiśniewski et al. [37]. Based on this number, candidate protein expression levels (Additional file 1: Figure S7a) were classified as follows: – not detected; + < 10,000/cell; ++ 10,000–100,000/cell; +++ > 100,000/cell. Similarity of protein expression profiles to RNA profiles was considered ‘NA’ (not applicable) when the protein was not detected, or only detected in few samples.

### SOMs

We created SOMs as implemented in the R package kohonen [92]. We started from a matrix of gene z-scores obtained from the RNA-Seq rlog-transformed data and selected all the DEGs according to the score *G* above. A supersom model was trained, considering seven different layers corresponding to the groups G01 to G07 above and a 20 × 20 toroidal hexagonal grid. The average codebook vector for the samples belonging to each group was plotted at each time point in order to obtain a topological map of the iTreg polarization over time.

### Signature scores

From the MSigDB [93] website (http://software.broadinstitute.org/gsea/msigdb/) the v5.1 C7 (‘immunologic signatures’) collection was downloaded and filtered to contain sets matching only ‘TH1’, ‘TH2’, ‘TH17’, ‘Treg’, ‘TCONV’ , or ‘CD4’ in their names. We incorporated four additional gene sets named ‘HUMAN_Treg_signature_UP’ , ‘HUMAN_Treg_signature_DN’ , ‘MOUSE_Treg_signature_UP’ , and ‘MOUSE_Treg_signature_DN’ , corresponding to the human and mouse up- or down-regulated genes in Treg versus Tcon as defined in Ferraro et al. [94] and Hill et al. [95], respectively. The human homologs of mouse genes were retrieved using the mouse Ensembl BioMart service. Then, for each pair of gene set (‘UP’ and ‘DN’) we calculated a score similarly to Gaublomme et al. [96]. Briefly, we started from a matrix of gene z-scores obtained from the rlog-transformed data. For each sample, we then defined the score as the mean of the genes in ‘UP’ minus the mean of the genes in ‘DN’. In order to quantify the relevance of each signature in a sample PC space, we calculated the Pearson correlation coefficient between each of the first three PC scores and the signature score. Finally, on a two-dimensional sample PCA plot we represented selected correlation value pairs (subset of those with *p* < 10^− 6^ in at least one of the three first PCs) as arrows starting from the origin and pointing to the pair of correlation values for the corresponding PC scores. We grouped the selected scores in three categories, i.e., ‘Treg vs. Tcon’ , ‘T cell activation’ , and ‘TGF-β treatment’.

### RNA:protein parallel comparison

We compared RNA and protein expression by matching RNA to the reference proteome, using the Ensembl gene ID as a key. We averaged log2Rs if multiple proteins mapped to the same gene. To obtain the heatmap in Fig. 3e, we first separately the obtained z-scores from the rlog-transformed RNA-Seq data and the log_2_R values for proteins, before combining them by columns. Hierarchical clustering was applied independently to the four blocks corresponding to ‘DEG and DEP’, ‘DEG not DEP’, ‘DEP not DEG’, and ‘not DEG and not DEP’. The DEG and DEP features were further classified into clusters, by cutting the tree in a number of partitions showing greater support, as evaluated by the average silhouette width and the Dunn Index recursively calculated for a range of 4–10 clusters. A Spearman correlation value was calculated for each gene:protein pair separately and values were grouped for each subset shown in Fig. 3e, where histograms show the corresponding distributions.

The subcellular localization was retrieved from the Human Protein Atlas [97] (www.proteinatlas.org). Only localizations with ‘Validated’ or ‘Supportive’ reliability were considered. The given localizations were classified in a reduced set, including Nuclear membrane, Nucleoli, Nucleoplasm, Cytoskeleton, Cytosol, Mitochondria, ER-Golgi, Plasma membrane, and Vesicles. For each group in Fig. 3e, the relative fractions of the categories were calculated. All the compartments for multi-localizing proteins were considered.

### Gene clustering and functional annotation

Gene clusters (Fig. 4) were identified using a model-based expectation-maximization algorithm, using the R package mclust [89]. We firstly extracted the genes differentially expressed in groups G03 and/or G05 (*G*_*G03*_ ≥ 2 and/or *G*_*G05*_ ≥ 2) from the matrix of the rlog-transformed data for the samples belonging to groups G02, G03, or G05. We then aggregated the individual donors by calculating an average value for each gene. The matrix was then z-score transformed. We estimated the number of Gaussian mixture components using the Bayesian Information Criterion calculated for a range of 2–50 components and for different parameterizations of the covariance matrix. We selected the model and the number of components maximizing the Bayesian Information Criterion. Then, mixture components were hierarchically combined as explained in Baudry et al. [98], resulting in the final selection of 42 clusters. The clusters were tested for functional enrichment with a hypergeometric test and categories from Gene Ontology (www.geneontology.org/), KEGG (http://www.genome.jp/kegg/), and Reactome databases (http://www.reactome.org/), using the topGO [99] with a ‘weight01’ algorithm, Category and ReactomePA libraries, respectively.

The association between the clusters was quantified by averaging the Spearman correlation coefficient calculated between all the pairs of genes from each cluster, and a *p* value was assigned to each association using a permutation approach. Briefly, for each combination of two clusters ***A*** and ***B*** with *n*_*a*_ and *n*_*b*_ genes and *m* ordered samples, we obtained the vector ***ρ*** of Spearman correlation coefficients between all *s* = *n*_*a*_ × *n*_*b*_ gene pairs; then, the correlation (*β*) between the two sets ***A*** and ***B*** was calculated as:

$$ \beta =\frac{\sum_{i=1}^s\rho i}{s} $$

To obtain a permutation-based *p* value, we repeated this procedure (*n*_*perm*_ = 10,000 times) by randomly labelling in each permutation the samples of cluster ***B***. As a result, we obtained a vector **π** with *n*_*perm*_ elements. We then calculated a *p* value as the fraction of the absolute values of **π** greater than the absolute value of the original *β* correlation value. We considered a significant association between every pair of clusters if *p* < 0.01.

### Transcriptional network reconstruction

To model the dynamics of the system, we reconstructed two gene networks using the log_2_(FPKM) values from ‘Early’ and ‘Late’ samples as indicated in Table 1 and only for the HEGs.

**Table 1:** List of samples and corresponding number of genes used to reverse-engineer the early and late network

	Time points	Groups	No. of samples	No. of genes
Early	T01, T02, T03	G01, G02, G03, G04, G05, G06	33	15,910
Late	T04, T05, T06	G02, G03, G04, G05, G06	45	15,910

We used ARACNe [100] to infer edges between the hubs and the expressed genes. Hubs were defined as the TFs that resulted differentially expressed at the gene (DEG) and protein (DEP) levels. In detail, we firstly selected the genes with *G*_*time*_ ≥ 2, *G*_*G03*_ ≥ 2, and/or *G*_*G05*_ ≥ 2, then from this list we selected the genes with *P*_*time*_ = 1, *P*_*G03*_ = 1, and/or *P*_*G05*_ = 1. RNAs and proteins were matched by the corresponding associated Ensembl gene ID. Finally, we considered all TF genes identified with either or both of two alternative annotations: (1) the human genes with a symbol annotated with the term ‘GO:0003700’ in the Gene Ontology Consortium database (www.geneontology.org) or (2) the Ensembl gene ID retrieved by querying the BioMart service (http://grch37.ensembl.org/) with the Gene Ontology ID ‘GO:0003700’. This list of 307 hubs was provided as input in the ARACNe run in order to calculate a MI value between a hub and all the other genes. Overall, 200 bootstrap networks were generated with a *p* value threshold of 10^− 10^ for MI estimation and a data processing inequality tolerance of 0.1 only for triplets involving at least one TF. A consensus network with a *p* value threshold of 10^− 8^ was finally obtained from all bootstrap networks previously generated. Early and Late consensus networks were imported into Cytoscape 3.4 (http://www.cytoscape.org/cy3.html) and they were compared with the DyNet algorithm [48] in order to identify the most rewired nodes, by ranking the nodes with a decreasing D_n_ score. To select a robust target core subnetwork of iTreg signature genes in the vicinity of FOXP3 (‘iTreg subnetwork’), we selected FOXP3 itself and all the nodes differentially expressed at the mRNA level in all iTreg conditions (*G*_*G03*_ ≥ 2 AND *G*_*G04*_ ≥ 2 AND *G*_*G05*_ ≥ 2 AND *G*_*G06*_ ≥ 2).

### DNase footprint networks

DNase-Seq data were obtained from ENCODE (http://hgdownload.cse.ucsc.edu/goldenPath/hg19/encodeDCC/wgEncodeUwDgf, sample ‘wgEncodeUwDgfTregwb78495824’) with DNase hypersensitivity (DHS) peaks. Protein-binding footprints (FP) within the DHS peaks were identified by scanning the DHS intervals for gaps in the signals using Wellington [101], with the parameters: -fp 6,40,2 -sh 4,30,2 -fdrlimit -10. The sequences of the resulting FP intervals were then scanned for protein binding motifs using the MatchTM program [102] with TRANSFAC positional weight matrices database [103], using parameters that minimize the sum of both positive and negative error rates. To improve prediction quality, several quality scores were calculated and examined, including FP *p* value, match score of the sequence to the motif, conservation scores (PhastCons and PhyloP in both glire and placental mammals), and footprint occupancy scores (FOS) [104], with a few modifications. First, the length of the flanking shoulders was optimized to yield the minimal FOS value; second, FOS was calculated for the predicted TF binding site within the footprint (termed ‘TFBS FOS’), as well as for the whole footprint (‘large FOS’). Networks were reconstructed by drawing an edge from the TF containing the binding motif to its target genes. A target gene is defined as such if binding is predicted to occur within its genomic coordinates, or in its promoter (1300 bp upstream).

### Enrichment for GWAS

The GWAS catalog was downloaded (www.ebi.ac.uk/gwas. Accessed 2016–08-14, version 1.0.1) and disease traits were grouped according to their parent term in the Experimental Factor Ontology, resulting in 17 categories. Two additional categories were added, Ai6 and Ai21, which include the GWAS genes for six common autoimmune diseases (MS, rheumatoid arthritis, T1D, CD, systemic lupus erythematous, and psoriasis) or the 21 autoimmune disorders as in Farh et al. [51], respectively. Each SNP was associated with the overlapping gene or the upstream and downstream genes as reported by the GWAS catalog after the Ensembl mapping pipeline. We tested the iTreg subnetwork for enrichment of the disease categories, with the full network as background, and ORs were calculated. For each category, a null distribution of ORs was obtained by resampling 10,000 sets of nodes with the same size as the iTreg subnetwork from the full network. The resampled OR null distribution was used to calculate a FWER using the step-down minimum *p* value procedure [105]. Corresponding one-tailed *p* values were obtained from the fraction of resampled runs with an OR greater than the original OR. This resampling-based *p* value (R) is similar to that obtained with a hypergeometric distribution (Additional file 6: Table S5). To test the enrichment for specific diseases, we considered the collection of diseases and their associated genes from Menche et al. [52]. ORs and *p* values were calculated with a hypergeometric test.

### Open Targets associations

To derive association scores from the Open Targets resource (www.opentargets.org) the version from September 2016 was used. A minimum score of 0.25 was used to filter out weak associations. The enrichment was tested as above with a hypergeometric test. The categories with a nominal *p* value below 0.05 were grouped by their therapeutic areas to draw a treemap using the treemap library. Diseases were included into all the therapeutic areas they belonged to.

### Detection of PPI modules

We considered data from three different public PPI data repositories (Human Interactome database [106], STRING v10.0 [107], and iRefIndex [108]), and integrated them into a large human PPI network with 26,655 nodes and 334,460 edges, which included both experimentally validated as well as computationally predicted interactions. We applied a score filter of 0.7 on the STRING interactions. To obtain an iTreg-specific PPI network (Treg-PPI), we filtered the integrated PPI network in a way that at least one end of the interacting nodes or their first neighbor is a DEP in iTregs (*p* < 0.05), and the interaction is being reported in at least two databases. We then applied a clustering method based on a simulation of stochastic flows on the graph, MCL [109], to identify topological modules in the Treg-PPI. We re-executed MCL with 10 different inflation parameters for detecting the modules with different sizes. We then mapped the categorized GWAS catalog genes described above and the core gene list (iTreg subnetwork) onto the Treg-PPI. Finally, we tested the identified modules for enrichment of core and disease genes using Fisher’s exact test (categories with nominal *p* < 0.05 were considered, and 119 hypotheses were tested). We did not consider the modules whose enrichment was exclusively due to core genes. The above data analysis pipeline was done in MATLAB and Cytoscape.

### Gene Set Enrichment Analysis (GSEA)

GSEA was performed using the package fgsea [110] and the iTreg subnetwork as gene set. We obtained gene lists from the Expression Atlas [111], contrasting the expression levels in controls and disease samples from datasets including MS and IBD patients (selected experiments: E-GEOD-12251, E-GEOD-14580, E-GEOD-16879, E-GEOD-23205, E-GEOD-4183, E-GEOD-60424, E-GEOD-6731, E-MEXP-2083, E-MTAB-2973, E-MTAB-69). We also included data from two recent publications [112, 113] not available in the Expression Atlas, which were processed to extract the DEGs between patients and controls. The genes were ranked by -log_10_P*sign(logFC) and the significance was assessed with 10,000 permutations.

### iTreg classification by LDA and RF

LDA [54] was performed to better understand the distribution of the data regarding different sets of features. LDA easily handles the case where the within-class frequencies are unequal. This method maximizes the ratio of between-class variance to the within-class variance in any particular dataset, thereby guaranteeing maximal separability. LDA was run on gene expression (rlog-transformed RNA-Seq) data.

For testing how well Mock-stimulated cells (G02) can be separated from iTregs generated by any protocol (G03-G06) by selecting two genes, LDA was run for all possible gene pairs selected from three different gene lists (‘37 candidate’ genes, ‘37 known’ Treg regulators, or ‘349 *iTreg*’, the latter being the 349 nodes in the iTreg subnetwork); we forced that both Mock-stimulated cells and iTregs should be presented in the cross-validation population. For confirmation, the same classifiers (all possible gene pairs from the three gene lists) were applied to rlog-transformed RNA data from the corresponding five time points (2, 6, 24, and 48 h, and 5 days) of the independent RNA-Seq dataset to classify iTregs (G04 + serum) versus Mock-stimulated cells (G02). We also defined ‘top classifiers’ as combinations of two candidate genes derived from the ‘37 candidate’ gene list, which separated groups 100% (in both runs per pair) in the Main dataset, and tested all these top classifier pairs in the independent dataset to determine the minimal accuracy per classifier pair for iTreg classification in the independent dataset. All calculations were performed using the classification tool box of MATLAB, with the auto regularization and five-fold cross-validation option. LDA results were further analyzed in GraphPad Prism (version 7) and Cytoscape.

As an unbiased way of feature (gene) selection to separate Mock (G02) and iTreg (G03–G06) conditions, we applied RFs to measure the importance of genes. RF is a nonparametric method that builds an ensemble model of decision trees from random subsets of features and bagged samples of the training data [114]. We ranked all 15,910 HEGs using the property PermutedVarDeltaError of TreeBagger class in MATLAB. All 37 candidate molecules, and all 37 known Treg regulators, were represented amongst HEGs. PermutedVarDeltaError returns the difference in the model error when permuting the values of a specific variable.

### Additional independent RNA-Seq dataset

#### Cell preparation

Naïve CD4+ T cells were isolated from PBMCs by negative selection using the naïve CD4+ T cell isolation kit II (Miltenyi Biotech; > 90% cell purity) from three healthy donors (male, age 32 years; female, age 28 years; male, age 28 years). The study was performed according to the Declaration of Helsinki and was approved by the ethics committee of the Ärztekammer Westfalen-Lippe and Medizinische Fakultät der Westfälischen Wilhelms-Universität Münster (registration number 2010262fS). The participants provided informed consent. Cells were stimulated with 0.5 μg/mL of plate-bound anti-human CD3 (clone UCHT1; Beckman Coulter) and 0.5 μg/mL of soluble anti-human CD28 antibody (clone CD28.2; eBioscience). Cells were stimulated in 24-well plates at 2 × 10^6^ cells/mL in 1 mL of X-Vivo 15 medium (Lonza). For Mock control cells, no further reagents were added, while for iTregs, 10 ng/mL of IL-2 (Peprotech), 10 ng/mL of TGF-β1 (R&D Systems), 10 nM ATRA (Sigma-Aldrich), and 10% (v/v) human serum (Human AB Serum, PAA Laboratories) were added. Samples were taken after 0.5, 1, 2, 6, 12, 24, 48, 72, 96, and 120 h, and lysates were stored at −80 °C until RNA extraction. At each time point, cells were stained by intracellular flow cytometry for FOXP3 expression.

#### Suppression assay

To assess the functional suppressive capacity of iTregs, suppression assays were performed as previously described for nTregs [115]. Briefly, PBMCs were isolated from fresh EDTA blood from a healthy donor and stained with cell proliferation dye efluor670 (eBioscience) according to the manufacturer’s instructions. These cells were re-suspended in X-Vivo15 (Lonza) and used as effector cells. iTregs or Mock control cells harvested on day 3, were washed and re-suspended in X-Vivo15. Effector cells were co-cultured in the absence or presence of different ratios of iTregs or Mock control cells (PBMC:iTreg/Mock cells 1:1 to 1:0.03). Proliferation of effector cells was stimulated with 0.5 μg/mL of anti-CD3 mAB (OKT3, Biolegend). On day 4, the proliferation of effector cells was determined with flow cytometry.

#### RNA-Seq

RNA was extracted with AllPrep RNA/DNA minikit (Qiagen) (RNA integrity number mean ± SD 9.1 ± 0.7996), mRNA was enriched via the poly-A tail using oligo-dT attached magnetic beads. Libraries were prepared according to Illumina TruSeq RNA Sample Preparation v2 Guide (part #15026495) and sequenced (paired-end with 2 × 100 bp read length) on an Illumina HiSeq 2500 instrument generating 6 to 37 Mio reads per sample. Paired-end reads were aligned with Tophat (version 2.0.8b) [116] and Bowtie2 (version 2.1.0) [117] to human reference genome (hg19) and Gencode transcriptome (v19). We estimated the mean insert size and standard deviation for each sample by mapping one million reads with Bowtie2. Read counts were determined using HTSeq-count (version 0.6.1) [118] with parameters ‘--stranded no --order name’.

## Additional files

Additional file 1: **Figure S1.** Flow cytometry and qRT-PCR quality control of cellular samples used for molecular profiling. **Figure S2.** Differential gene and protein expression analysis and overlap of RNA-Seq and proteomics data. **Figure S3.** Exploratory analysis of RNA-Seq and proteomics data. **Figure S4.** Estimated number of proteins per cell based on iTreg proteomics data. **Figure S5.** Gene Ontology and pathway enrichment analysis of the DEG and DEP clusters. **Figure S6.** iTreg subnetwork reconstruction strategy. **Figure S7.** Features of the iTreg candidate molecules and the confirmatory independent RNA-Seq dataset. **Figure S8.** Linear Discriminant and Random Forest analyses confirm the potential of candidate genes to classify iTregs. **Figure S9.** Experimental validation of novel FOXP3+ Treg regulatory molecules. (PDF 3722 kb)

Additional file 2: **Table S1.** Signature scores. (XLS 30 kb)

Additional file 3: **Table S2.** Differentially expressed genes and proteins. (XLSX 6515 kb)

Additional file 4: **Table S3.** Clustering and functional annotation. (XLSX 214 kb)

Additional file 5: **Table S4.** Network nodes and edges. (XLSX 109 kb)

Additional file 6: **Table S5.** Functional and disease annotation iTreg subnetwork. (XLSX 37 kb)

Additional file 7: **Table S6.** Random Forest ranking for iTreg classification. (TXT 546 kb)

Additional file 8: **Table S7.** shRNA clone list. (XLSX 15 kb)
