# Supplementary material for: Time-resolved transcriptome and proteome landscape of human regulatory T cell (Treg) differentiation reveals novel regulators of FOXP3
Source: BMC Biol. 2018 May 7;16:47. doi: 10.1186/s12915-018-0518-3 (PMC5937035; doi:10.1186/s12915-018-0518-3)

SUPPLEMENTARY FIGURES 1-9 AND LEGENDS

Supplementary Figure 1. Flow cytometry and qRT-PCR quality control of cellular samples used for molecular profiling. (**a**) Naïve human CD4+ T cells were magnetically negatively isolated using the Naïve CD4+ T Cell Isolation Kit II, human, and the purity is shown. Right panel: *ex vivo* Tregs ("nTreg") from the corresponding donors. (**b-d**) Human naïve CD4+ T cells were cultured as in Fig. 1 (**b**) Gating strategy for flow cytometric analysis of iTreg cultures (shown is an iTreg sample cultured for 6d with TGF-β + ATRA and re-stimulated with PMA/Ionomycin/Brefeldin A. (**c**) Flow cytometry as in Fig. 1b-c, but without PMA/Ionomycin restimulation. (**d**) An aliquot of the RNA extract from molecular profiling samples (filled symbols) was used for qRT-PCR. *FOXP3* mRNA (primers binding exon 12) and *IKZF4* mRNA was quantified by SYBR Green and Taqman assay respectively, normalized to *RPL13A* expression. Fold change was calculated compared to unstimulated naïve T cells from the corresponding donor (set to 1). Open symbols: additional verification donor. Lines: mean values of all four donors. (**e**) Western Blot analysis of FOXP3 expression. Samples were processed in the same way as samples for molecular profiling in (a-d), but are from independent donors and experiments. Left: Blots from samples of one representative donor. Middle: Summary of Western blot data from 3 donors (each symbol represents one donor); bars represent Mean + SEM. Total FOXP3 expression was quantified, fold change compared to Mock stimulated cells (set to 1) was calculated within each donor (one donor per gel), and data were normalized to expression levels of the PARP protein. Right: Fraction of FOXP3+ cells in the corresponding donors (symbols as in middle panel) is shown for comparison, determined by flow cytometry and gated on live CD4+ cells as in (b).

Supplementary Figure 2. Differential gene and protein expression analysis and overlap of RNA-Seq and proteomics data. (**a-b**) Overlap of RNA-Seq and proteomics data, based on Ensembl gene IDs, was analyzed and is displayed as Venn diagrams; grey shading and size is proportional to fraction size. (**a**) All quantified proteins (*i.e.* those detected in each individual sample) were compared to all detected protein-

coding genes. The number of genes in each section is indicated, and the fraction of the sum of genes is given in percent. (**b**) All quantified proteins were compared to all highly expressed protein-coding genes. (**c-e**) Differentially expressed genes (DEGs) were derived by modeling differential gene expression over time, with 3 different methods: DESeq2 with time as a factor (F), DESeq2 with time as a spline (S), or with maSigPro. A list of differentially expressed proteins (DEPs) was obtained from Limma analysis. DEG or DEP time designate genes/proteins that are differentially expressed over time (activation effect). DEG/DEP for the indicated iTreg groups indicate differential expression compared to the Mock stimulated control group. (**c**) Summarization of genes significantly changed. In DESeq2, a gene was considered significant if the adjusted p<0.01 for any coefficient of the coefficient group (*i.e.* the coefficient for the group and the all the corresponding interaction coefficients (group x time point)). maSigPro: A gene was considered significant if the polynomial fit was significant (p.adj<0.01) and R2>0.7, considering coefficient groups. A gene was called "DEG" if significant with at least 2 of the 3 methods. (**d**) Summarization of DEP with the Limma method (FDR<0.05), based on Ensembl protein ID. (**e**) DEPs and DEGs differentially expressed in any comparison (over time or in any iTreg condition compared to Mock control stimulated cells) were defined. Only protein-coding genes were considered for calculating the overlap with DEPs.

Supplementary Figure 3. Exploratory analysis of RNA-Seq and proteomics data. (**a,d**) A sample-to-sample correlation heatmap is shown. Blind regularized logarithmic transformed values (rlog) or relative quantification values ($\log_2$R) were used for RNA-Seq (a) and proteomics data (d), respectively. One minus Pearson correlation was used as distance. Correlation blocks were isolated by splitting the dendrogram and they mostly separate different time points. (**b,e**) Principal Component (PC) analysis on rlog RNA-Seq (b) or $\log_2$R proteomics (e) data. PC1 and PC2 separate samples based on the time point (grey scale) and group (label; G01: unstimulated Tnaïve; G02: Mock stim.; G03: iTreg TGF-β; G04: iTreg TGF-β + ATRA; G05: iTreg TGF-β + ATRA + Rapa; G06: iTreg TGF-β + butyrate; G07: nTreg). (**c, f**) A separate linear model was fitted between each of the PC scores and a series of biological or

technical explanatory variables, for RNA-Seq (c) or proteomics (f) respectively. The R^2 was calculated for each of the models and values were plotted using a heatmap, in order to assess associations between the PCs and the explanatory variables.

Supplementary Figure 4. Estimated number of proteins per cell based on iTreg proteomics data. Calculation of the estimated number of proteins per cell from proteomics data according to Wiśniewski JR *et al.*, Molecular & Cellular Proteomics 2014. Values were averaged for all the used T cell samples before calculation; note that samples in which the respective protein was not detected were not considered in calculating the average. For information on detection of proteins in individual samples, see Additional_File_3_Table_S2 containing the complete proteomics ratio data of all identified proteins. Several signature proteins and regulators well-known for Tregs and/or other T cells are highlighted in red, and their gene name is indicated. "Housekeeping" proteins (beta Tubulin and RPL13A) are highlighted for comparison in blue.

Supplementary Figure 5. Gene Ontology and pathway enrichment analysis of the DEG&DEP clusters. The DEG&DEP clusters (*a-e* in Figure 3e) were tested for enrichment of GO Biological Process, GO Cellular Component and Reactome pathways. The clusterProfiler library was used to calculate the hypergeometric enrichment. Up to five categories are shown per cluster and the number of genes per cluster is shown in parentheses. GeneRatio: the fraction of genes in the cluster that are enriched for the indicated category; p.adjust: Benjamini & Hochberg adjusted p-values.

Supplementary Figure 6. *iTreg subnetwork* reconstruction strategy. (**a**) We obtained a score (left panel) for the "FOXP3 known regulator signature" (see *Methods*). The right panel shows corresponding gene levels (rlog z-score). (**b**) HEGs were extracted, and early (T01-T03; 0h, 2h, 6h) and late (T04-T06; 24h 48h, 6d) expression values were separated in order to reconstruct a co-expression network. Groups G01 - G07 as in Fig. 2 (**c**) We used ARACNe to infer edges between the hubs and the expressed genes

(see *Methods*). Early and Late consensus networks were compared with the Cytoscape DyNet algorithm in order to identify the most rewired nodes. A union network ("*Full network*") was obtained and the nodes were ranked with a decreasing rewiring score. To obtain the "*iTreg subnetwork*", we selected FOXP3 itself and all the nodes differentially expressed at the mRNA level in all iTreg conditions (G03 to G06 *vs.* G02). (**d**) Expression of all 349 *iTreg subnetwork* genes is shown; labels as in Fig. 7a. Values were row-scaled and -clustered (Euclidian distance complete linkage). (**e**) Gene clusters (left column, as in Fig. 4) containing ≥10 *iTreg subnetwork* genes are listed (exact number in the respective cluster, based on gene symbol, is indicated in the middle column). Right column: expression profile of the gene cluster, as in Fig. 4. (**f**) DEGs were determined between human primary pre-activated CD45RA+CD25-CD4+ T cells transduced with human FOXP3 *versus* with corresponding empty vector (EV) from Dhuban *et al. (Sci. Immunol.* 2017). GEO2R default settings (submitter-supplied) were used and DEGs defined as significant if Benjamini & Hochberg adjusted p-value (FDR)<0.05. Retrieved RefSeq IDs of significant DEGs were converted to ENSG IDs, and the Venn diagram shows overlap with *iTreg subnetwork* genes. Corresponding gene symbols from the shared genes are indicated in grey.

Supplementary Figure 7. iTreg candidate molecules and confirmatory independent RNA-Seq data set. (**a**) Features of candidate molecules (see *Results* and *Methods*). For PLEC gene ID, data from 2 protein IDs were pooled. Annotation as TF based on the Gene Ontology Consortium ("GO") and/or BioMart services is given ("++" if both). Disease association scores based on Open Targets database is indicated for IBD, MS, and immune system disease ("ISD"). (**b**) Control list of 37 known Treg regulators (Treg up genes/positive regulators ("pos") or Treg down genes/negative regulators ("neg")). All genes in (a) and (b) are DEGs in ≥1 iTreg condition compared to Mock stimulation; "DEG all iTregs" indicates genes DEGs in all 4 iTreg groups. (**c**) FOXP3 (upper) and CD25 (lower panel) expression by flow cytometry for the independent RNA-Seq data set from Fig. 7c. Individual donors are shown (n=3). Black: Mock stim. cells (anti-CD3/anti-CD28, no IL-2); blue: iTregs (TGF-β + ATRA + IL-2 + serum). (**d**) Suppression assay of iTregs or Mock cells induced for 3 days as in (c), and used as suppressor cells

(“Tsup”) in a 0.03-1 ratio towards proliferation dye efluor670-labeled effector PBMCs (“Eff”). Co-cultures were stimulated with anti-CD3 antibody. After 4 days, effector cell proliferation was determined, gated on CD4+ effector T cells (“CD4+TEff”, circles) or “CD8+TEff” (squares). Percent suppression by iTregs (blue) or Mock stim. cells (black, grey) is shown (mean ± SEM, n=5 donors). (**e**) Comparison of the Main (triangles) and independent (circles) RNA-Seq data sets for treatment Group 02 (G02), Mock stim. cells (-/+ IL-2) and treatment Group 04 (G04), iTregs induced with TGF-β + ATRA + IL-2 (-/+ serum). PCA was performed after batch correction with an Empirical Bayes method (ComBat). PC1 and PC2 are displayed; ellipses are the 95% normal probability for the corresponding time points. n=3 donors per data set.

Supplementary Figure 8. Linear Discriminant and Random Forest analysis confirms the potential of candidate genes to classify iTregs. (**a-c**) Linear discrimination was performed on the Main and independent data sets as in Fig. 7d. All pairs of the “37 candidate” gene list were tested in LDA in the Main data set, and “top classifiers” performing 100% (in both runs per pair) were tested regarding their potential for group separation in the independent data set that was not used for candidate selection. (a) displays all the gene pairs separating iTreg and Mock stimulation groups with 100% accuracy in the Main data set; gene pairs are connected by an edge which is colored based on performance (minimal % accuracy of 2 runs per pair) of the pair in the independent data set. (b) shows these accuracy values for group separation in the independent data set for the same pairs as in (a) as boxplot (Whiskers: min. to max. value; +: mean value; n = 76 pairs). (c) scatter plot of expression (rlog-transformed counts) of one example pair among top classifier candidate gene pairs (73% accuracy in the independent data set; see also Fig. 7e for its analysis in the Main data set). (**d**) Random Forest (RF) analysis was performed to rank all individual 15910 HEGs for their importance in distinguishing iTregs (G03-G06) from Mock stimulated cells (G02) in the Main data set. The top-ranking 20 genes (indicated in bold and separated by blue line), along with all additional candidate molecules as well as FOXP3 are shown. The RF rank is given in the right column and top-100 ranking candidate genes are highlighted according to the given red

color scale. Black boxes indicate whether a gene belongs to the “37 candidates” gene list or “37 known” gene list.

Supplementary Figure 9. Experimental validation of novel FOXP3+ Treg regulatory molecules. (**a**) Primary naïve CD4+ T cells were transduced as in Fig. 8 with individual or pooled shRNAs targeting CD4, or shScr control. Cells were subsequently stimulated for 5 days under Mock differentiation conditions (in the presence of puromycin). Cells were surface-stained with anti-CD4 antibody, followed by fixable viability dye staining and fixation with 2% PFA. CD4 expression was monitored by flow cytometry, pre-gated on live cells. Left panel: representative donor transduced with the indicated shRNAs. Right panel: median fluorescence intensity (MFI) for CD4 was normalized to shScr (set to 100%) within each donor. Displayed are mean ± SEM values of normalized MFI (nMFI), each dot represents an individual T cell donor (n=2-5 donors from 2 independent experiments). Significance of CD4 knockdown *versus* shScr was calculated by one-sample *t* test compared to the hypothetical value 100. p-value labels: ns: not significant; *: p<0.05; ***: p<0.001, ****: p<0.0001. CD4-targeting shRNA clone labels: shCD4 #1:TRCN0000057613; shCD4 #2:TRCN0000057614; shCD4 #3:TRCN0000057615; shCD4 #4:TRCN0000057616; shCD4 #5:TRCN0000057617. (**b**) Transduction efficiency is shown, using a pLKO.1 plasmid in which the puromycin resistance gene was replaced by GFP (pLKO3G). Primary naïve CD4+ T cells were transduced as in Fig. 8, and after 5 days of stimulation with anti-CD3, anti-CD28 and IL-2 (without puromycin), cells were stained with fixable viability dye and fixed with 2% PFA. GFP expression was measured by flow cytometry, gated on live cells. Cells transduced with pLKO.1-shScr are indicated in black/grey, cells transduced with pLKO3G are depicted in green (3 different lines represent 3 different T cell donors). Transduction efficiency was 50-65% based on GFP expression. (**c, d**) T cells were transduced with shRNA targeting candidate genes or controls as in Fig. 8, but under Mock stimulation instead of iTreg differentiation. Analysis and display as in Fig. 8.

Supplementary Figure 1

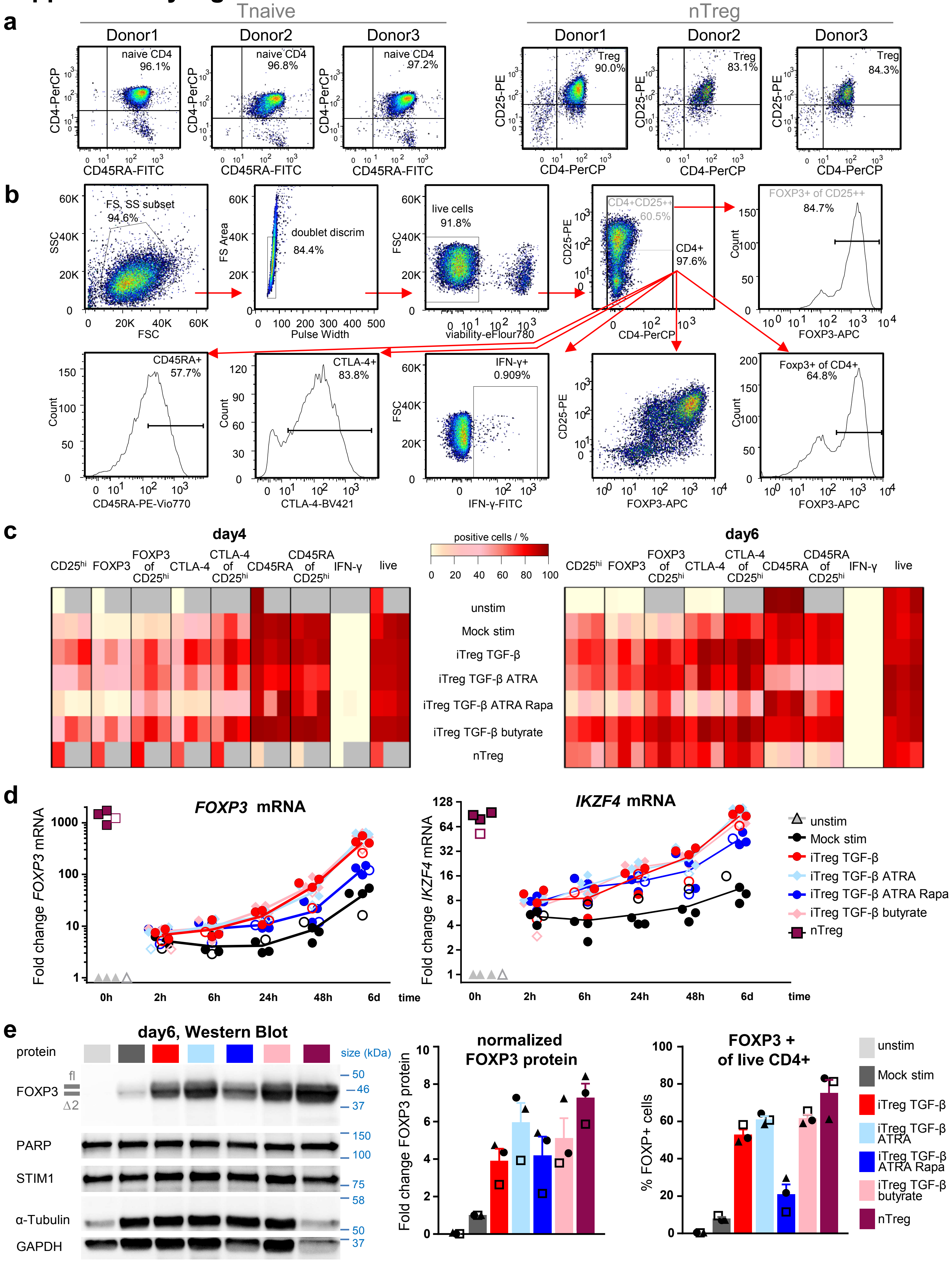

Supplementary Figure 2

a

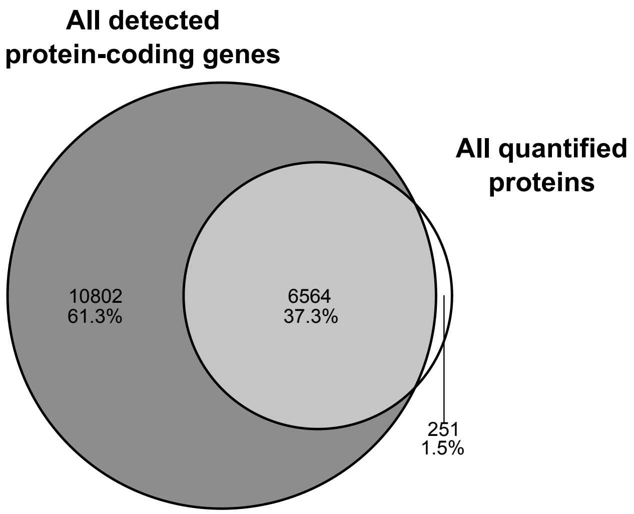

b

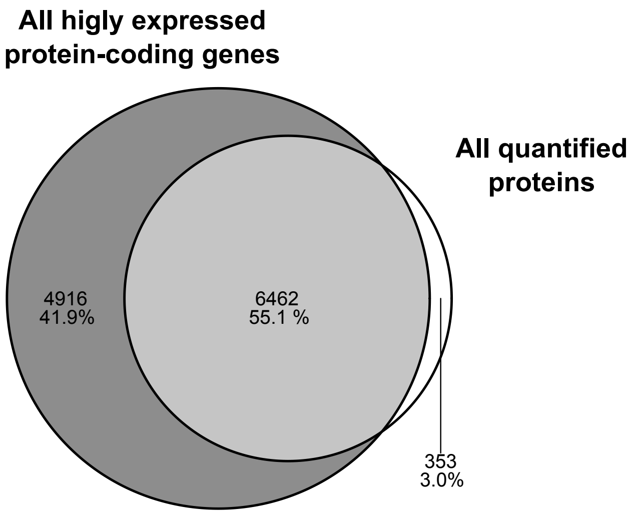

c

Method	differentialy expressed (Time)	differentialy expressed (iTreg TGF-β)	differentialy expressed (iTreg TGF-β ATRA)	differentialy expressed (iTreg TGF-β ATRA Rapa)	differentialy expressed (iTreg TGF-β butyrate)
maSigPro	**3340**	**1723**	**1695**	**2373**	**1733**
DESeq2 (F)	**11308**	**2148**	**1724**	**1905**	**2862**
DESeq2 (S)	**10896**	**1164**	**1375**	**2290**	**3141**
DEG all 3 methods	**2962**	**307**	**305**	**481**	**351**
DEG ≥2 methods	**10093**	**1314**	**1279**	**1725**	**2249**
DEG ≥2 methods; protein-coding	**8911**	**1243**	**1177**	**1596**	**2129**

d

Method	differentialy expressed (Time)	differentialy expressed (iTreg TGF-β)		differentialy expressed (iTreg TGF-β ATRA Rapa)	
DEP Limma	**4660**	**614**		**1689**	

e

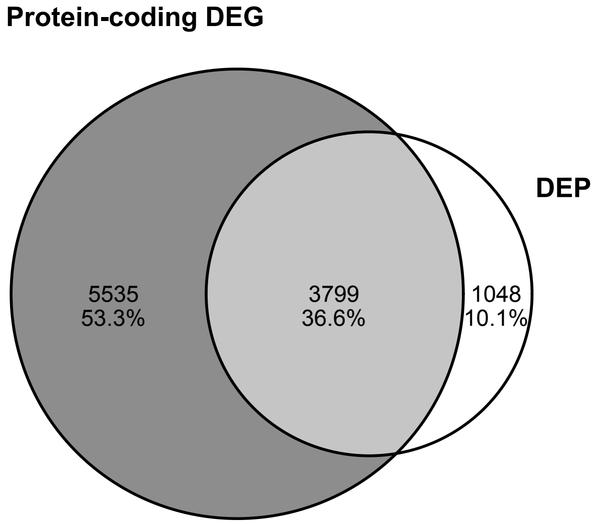

Supplementary Figure 3

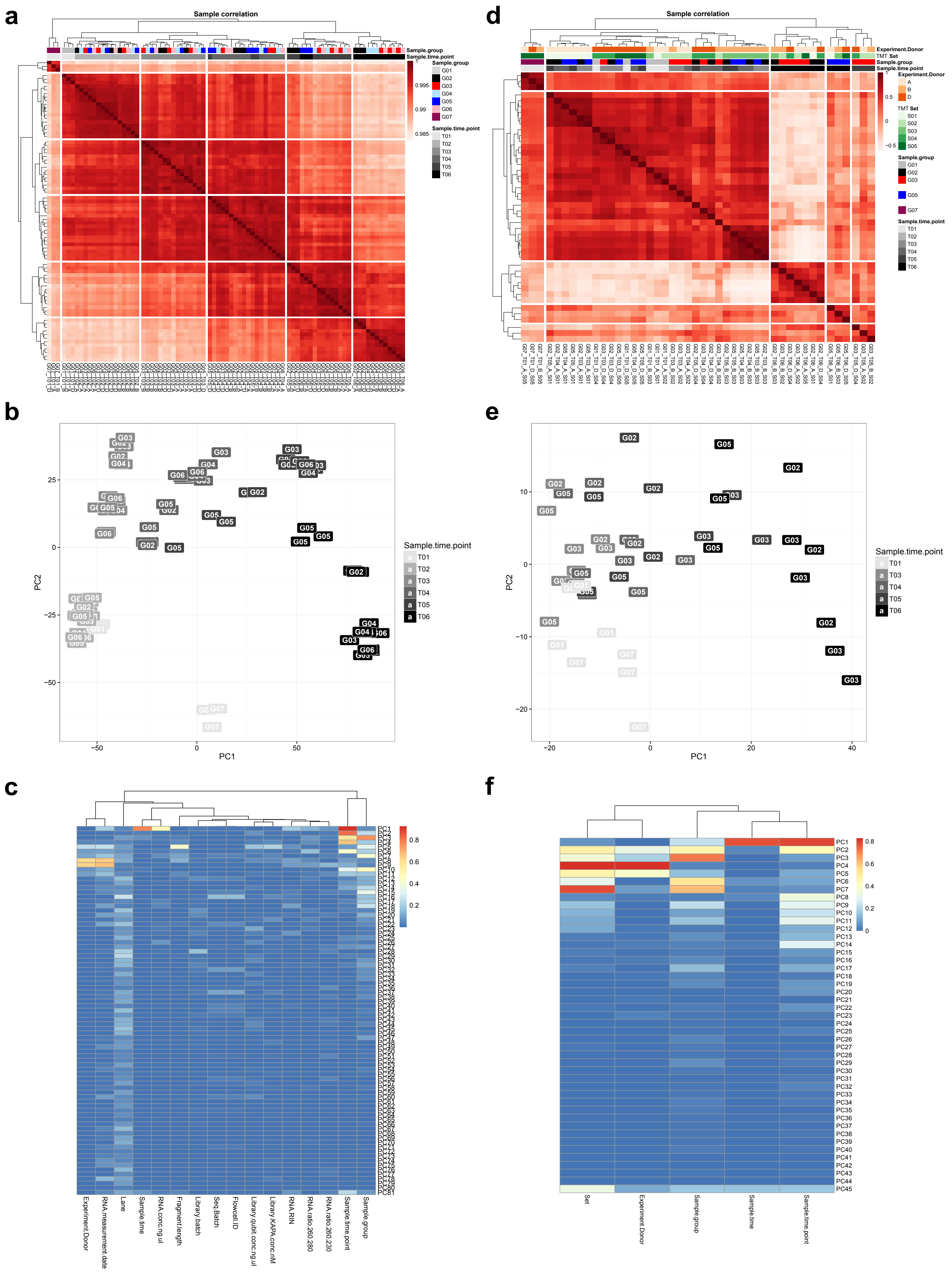

Supplementary Figure 4

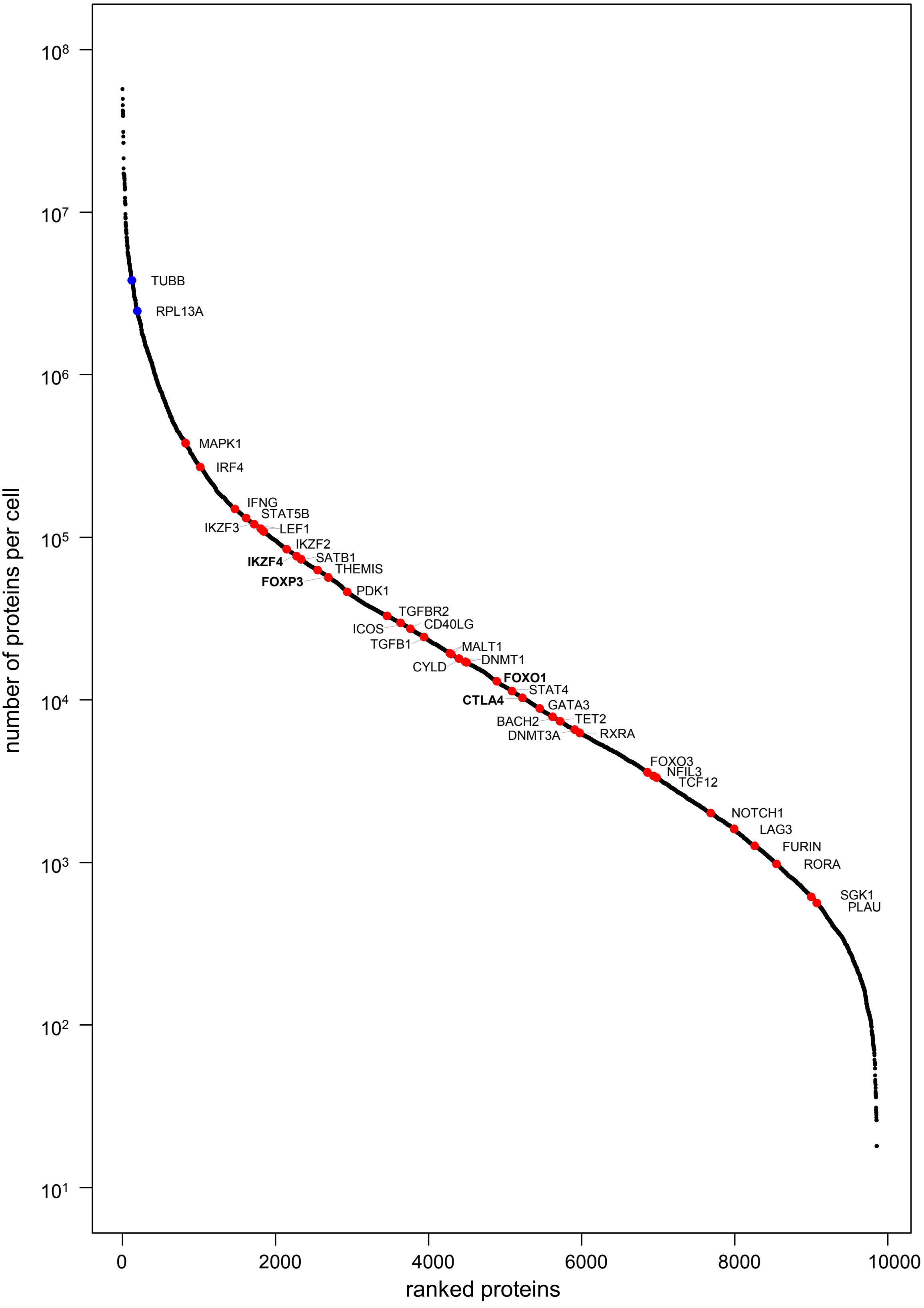

Supplementary Figure 5

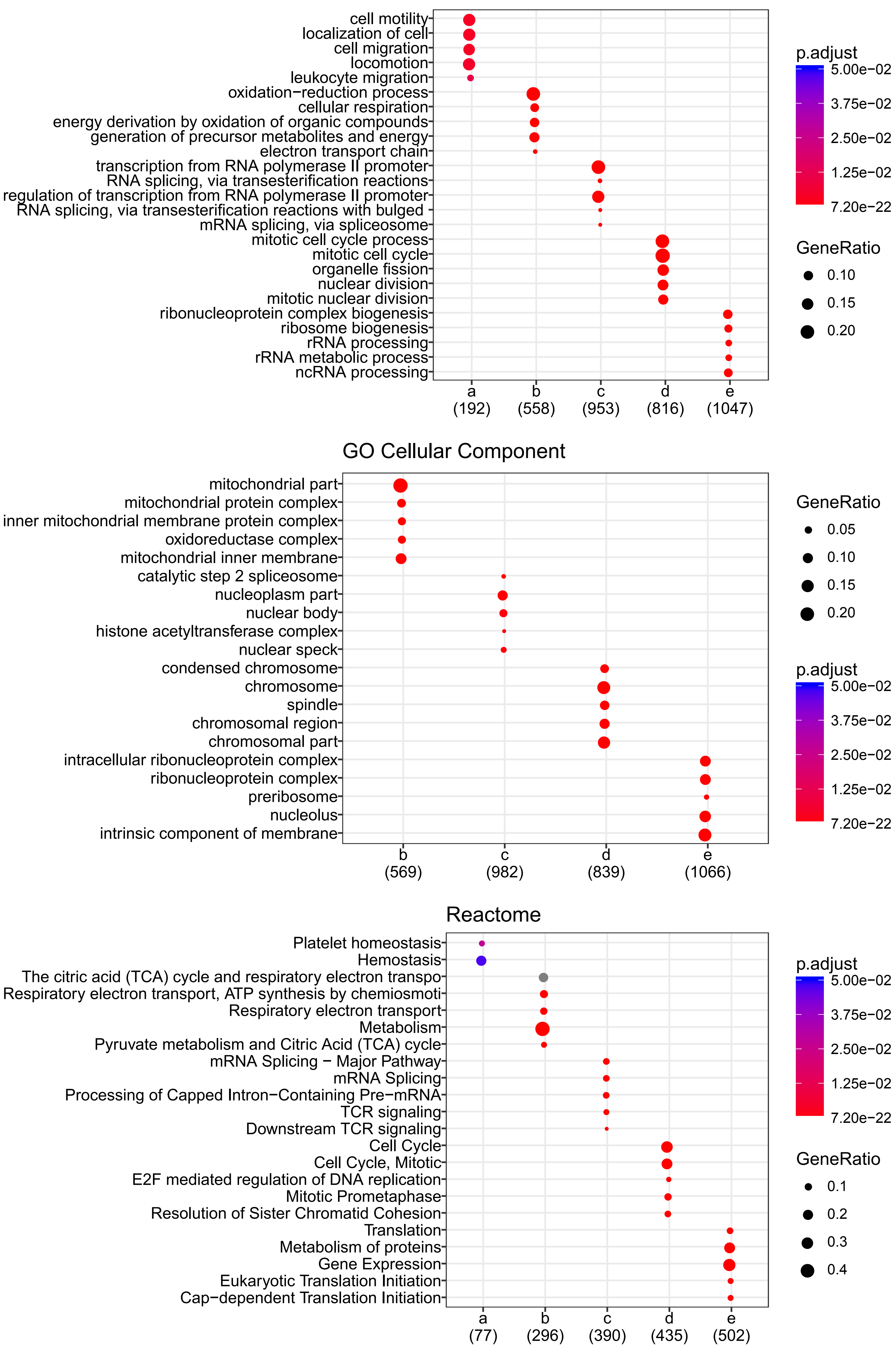

Supplementary Figure 6

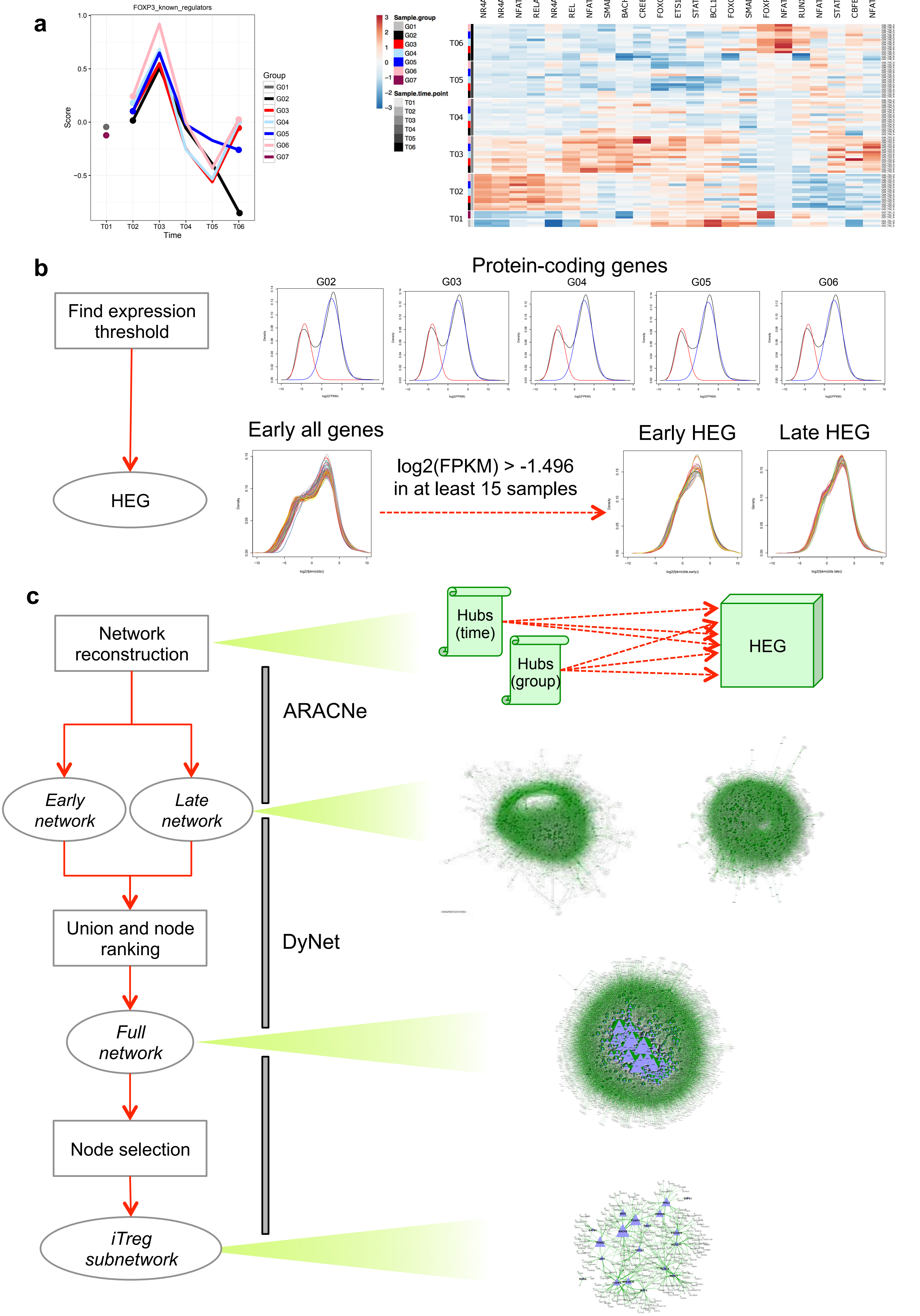

Supplementary Figure 6 – continued

d

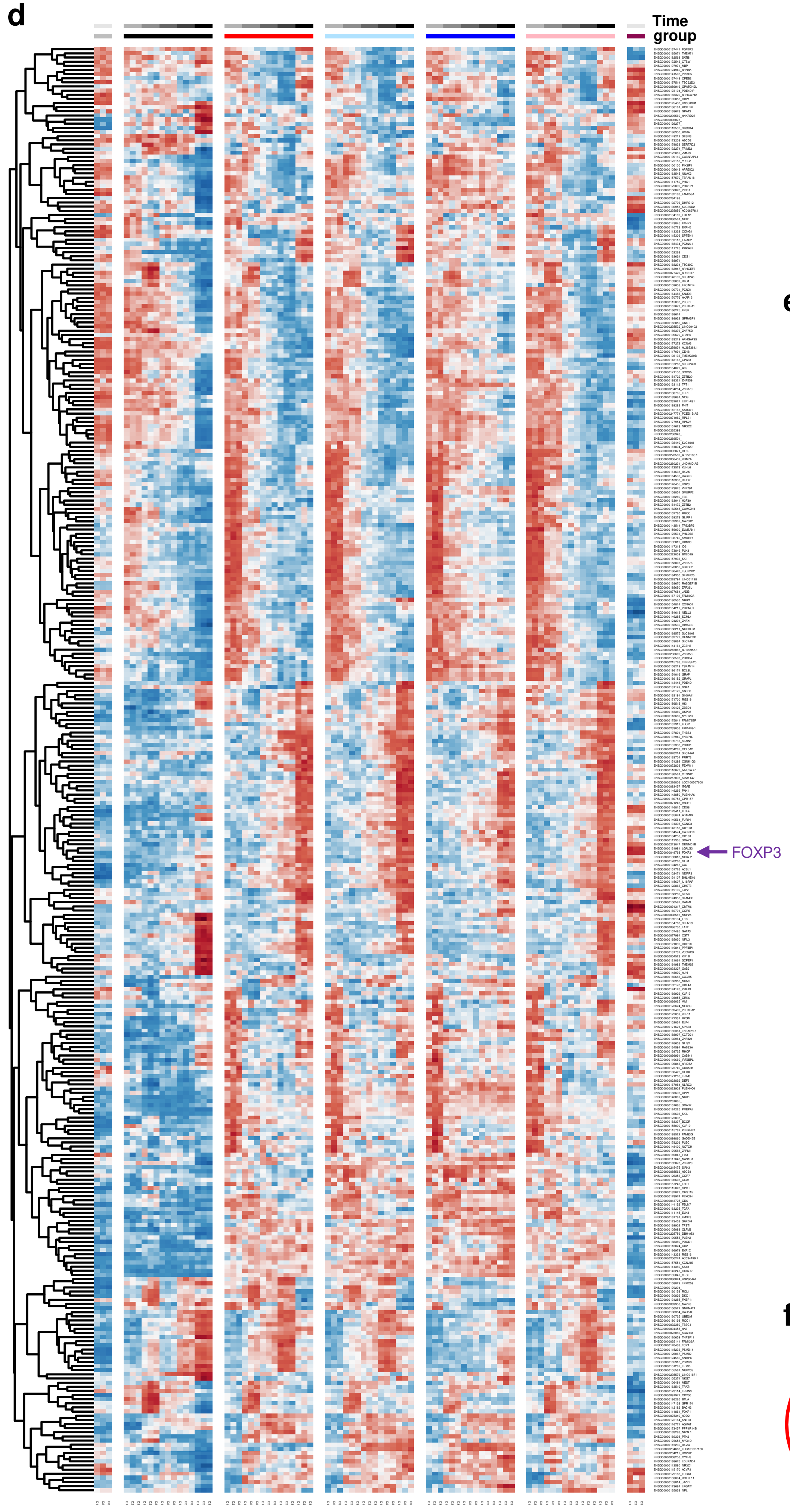

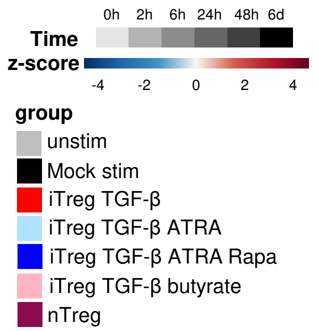

e

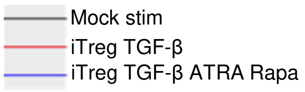

Cluster #	Number of iTreg subnetwork genes	Expression profile
3 (FOXP3)	61	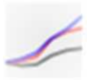
10	32	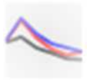
11	14	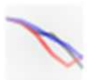
15	15	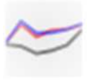
23	17	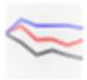
25	14	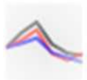
28	10	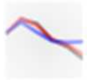
29	13	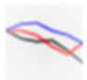
35	21	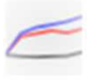
38	10	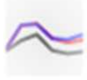
42	14	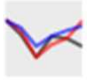

f

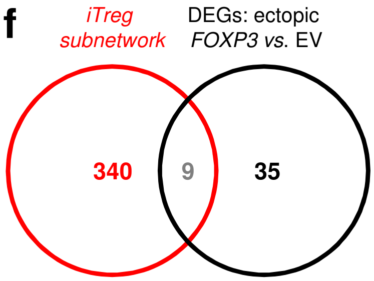

9 shared genes:

FOXP3
PIK3IP1
OLFM2
HS3ST3B1
SLC35D2
SESN3
BCL2L11
FAM102A
IL13

Supplementary Figure 7

a 37 candidates

Candidate Gene	Gene expression level	Protein expression level	DEG all iTregs	Protein similar to RNA	iTreg hub	TF (GO and/or BioMart)	Open Targets_IBD	Open Targets_MS	Open Targets_ISD
ATP1B1	+ / ++	++	+	+	-	--		0.02	0.12
BCOR	+++	+	+	+	-	--			
BHLHE40	++ / +++	+	+	+	-	++			0.27
CA2	+	++	+	+	-	--	0.14	0.11	0.20
CD58	+	-	+	NA	-	--	0.06	0.87	0.88
CHST15	+ / ++	-	+	NA	-	--	0.13		0.13
CTSL	++ / +++	+++	+	+	-	--	0.03	0.02	0.17
CYTH3	++	-	+	NA	-	--			0.04
ELF4	++	+	+	+	-	++			0.24
ELK3	++ / +++	+	+	+	-	++	0.11	0.21	0.25
EVA1C	+ / ++	-	+	NA	-	--			
KCNJ15	+	-	+	NA	-	--	0.05		0.17
KCTD21	+ / ++	+	+	+	-	--			
KLHL25	+ / ++	+	+	+	-	--			
LDLRAD4	++ / +++	+	+	+	-	--			0.08
LGALS3	+ / +++	+++	+	+	-	--	0.07	0.19	0.33
MAFG	++	++	-	+	+	++			0.20
MID2	++	+	+	+	+	+-			
MYL12B	+++	-	+	NA	-	--		0.31	0.35
NKD1	+ / ++	-	+	NA	-	--			0.02
NR3C1	+++	++	+	+	+	++	1.00	1.00	1.00
OCIAD2	++	+++	+	+	-	--			0.03
OLFM2	++	+	+	+	-	--			
PLEC	+++	+	+	+	-	--	0.07	0.02	0.09
PLEKHO1	++ / +++	++	+	+	-	--	0.02		0.15
PMEPA1	+ / +++	-	+	NA	-	--	0.07		0.07
PTK2	++ / +++	+	+	+	-	--			0.07
RGCC	++ / +++	++	+	+	+	+-	0.15	0.04	0.16
RGS16	+ / +++	+	+	+	-	--			0.16
S100A11	++ / +++	+++	+	+	-	--	0.09	0.20	0.24
SARDH	++	+	+	+	-	--			
SKIL	++ / +++	++	+	-	-	+-			0.06
TGFA	+ / ++	-	+	NA	-	--	0.04		0.21
TRIM22	+++	++	+	+	+	++	0.16	0.43	0.48
USP35	+ / ++	-	+	NA	-	--			
ZNF629	+ / ++	++	+	+	+	+-			
ZNF821	+ / ++	-	+	NA	-	--			

b 37 known

Known Treg regulator	DEG all iTregs	iTreg hub	TF (GO and/or BioMart)	Role in Tregs	Open Targets_IBD	Open Targets_MS	Open Targets_ISD
BACH2	+	+	++	neg	0.62	0.32	0.80
CCR5	+	-	--	pos	0.06	0.08	1.00
CD40LG	-	-	--	neg	0.08	0.06	1.00
CTLA4	-	-	--	pos	0.11	0.06	1.00
CYLD	-	+	+-	neg	0.02	0.19	0.29
DNMT1	-	-	--	neg	0.04	0.20	0.29
DNMT3A	-	-	--	neg			0.47
FOXO1	-	-	++	pos	0.65		0.68
FOXO3	-	-	++	pos	0.05	0.07	0.30
FOXP3	-	-	++	pos	0.09	0.05	1.00
FURIN	+	-	--	pos			0.12
GATA3	+	+	-+	pos	0.04	0.19	0.58
ICOS	-	-	--	pos	0.05	0.05	1.00
IFNG	-	-	--	neg	0.76	0.07	0.84
IKZF2	-	-	--	pos			0.02
IKZF3	-	+	++	pos	0.86	0.05	0.95
IKZF4	+	+	++	pos			0.88
IRF4	-	-	++	pos	0.38		0.53
LAG3	-	-	--	pos		0.04	0.30
LEF1	+	+	++	neg	0.06	0.31	0.40
MALT1	-	-	+-	pos	0.02	0.38	1.00
MAPK1	-	+	+-	pos		0.36	0.38
NFIL3	+	+	++	pos	0.15	0.01	0.28
NOTCH1	+	-	-+	pos	0.87	0.02	0.92
PDK1	-	-	--	pos			0.05
PLAU	-	-	--	pos	0.68	0.04	0.69
RORA	-	-	++	neg	0.05		0.26
RXRA	+	+	++	pos	0.00	0.02	1.00
SATB1	+	+	++	neg	0.61	0.25	0.72
SGK1	-	-	+-	neg		0.18	0.22
STAT4	-	+	++	neg	0.78	0.04	1.00
STAT5B	-	-	++	pos			1.00
TCF12	-	+	++	neg			0.28
TET2	-	-	--	pos	0.60	0.03	0.67
TGFB1	-	-	--	pos	0.10	0.27	0.36
TGFBR2	-	-	--	pos		0.26	0.30
THEMIS	-	-	--	neg/pos	0.55	0.28	0.65

c

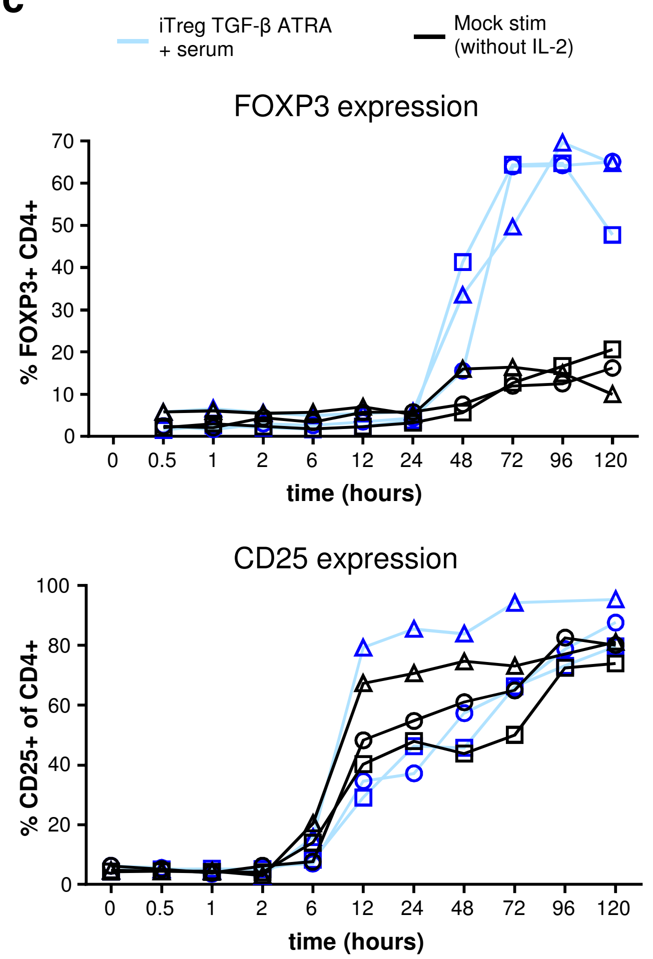

d

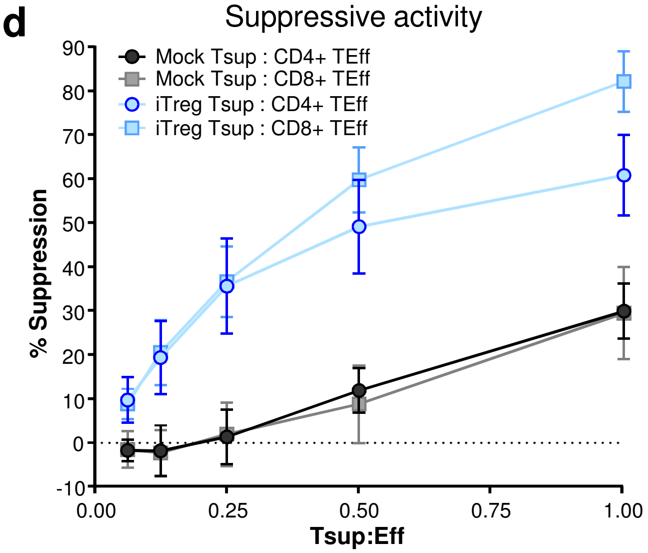

e

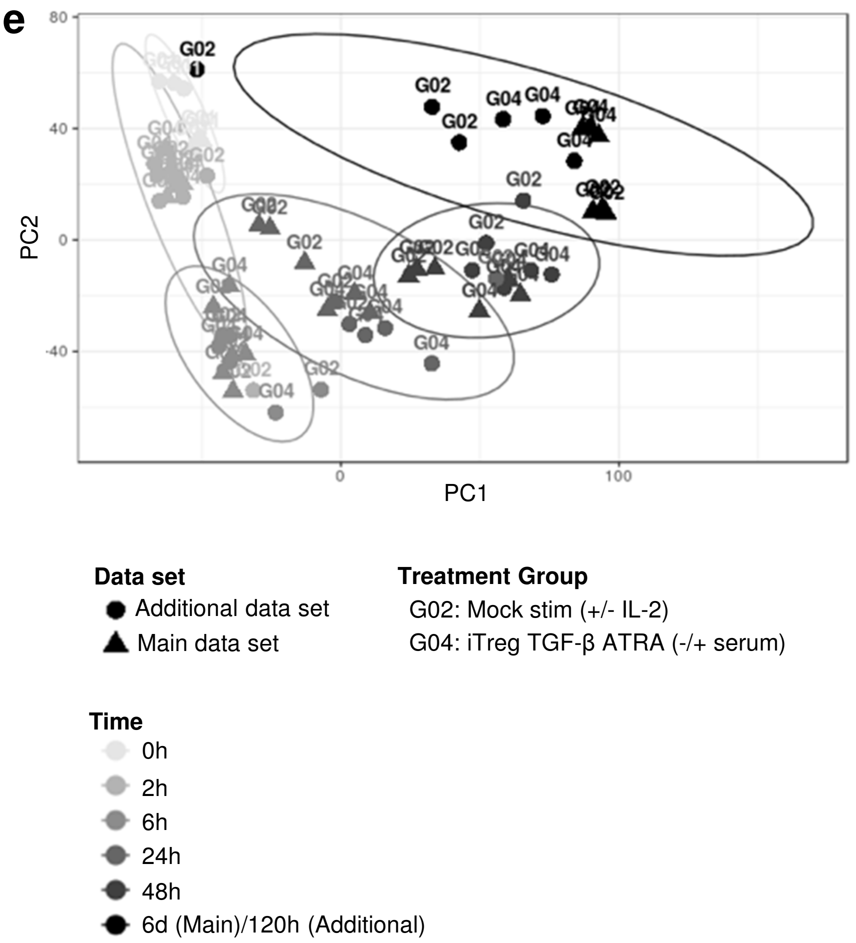

Supplementary Figure 8

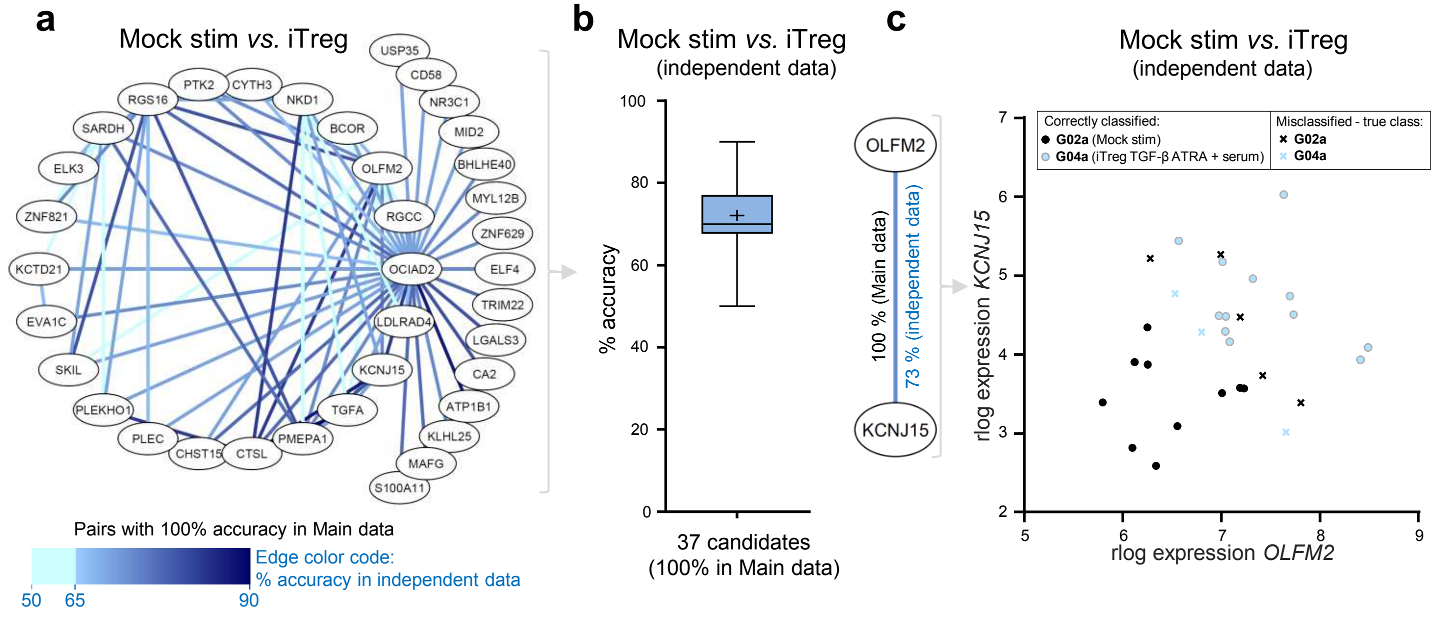

d

Known Treg	Candidate	Gene name	Random Forest Rank
	■	KCTD21	1
	■	OCIAD2	2
		SMAD7	3
	■	CTSL	4
■		**IKZF4**	5
		N/A (lncRNA)	6
	■	CHST15	7
■		FURIN	8
		CXCR5	9
	■	SARDH	10
	■	BCOR	11
	■	OLFM2	12
		QPCT	13
		RP3-330M21.5	14
	■	EVA1C	15
		ERBB2IP	16
	■	KLHL25	17
	■	LDLRAD4	18
		BPGM	19
		DBH-AS1	20
	■	USP35	24
	■	MYL12B	34
	■	NKD1	36
	■	PMEPA1	40
	■	TGFA	42
	■	CYTH3	46
	■	ELK3	47
	■	PLEKHO1	51
	■	SKIL	55
	■	KCNJ15	58
	■	CA2	85
■		**FOXP3**	101
	■	PTK2	865
	■	NR3C1	1297
	■	CD58	3635
	■	ZNF629	6101
	■	ELF4	6217
	■	S100A11	7408
	■	MID2	8182
	■	ATP1B1	9690
	■	BHLHE40	10632
	■	LGALS3	10750
	■	RGS16	10854
	■	PLEC	11080
	■	RGCC	13520
	■	MAFG	13946
	■	ZNF821	14521
	■	TRIM22	15563

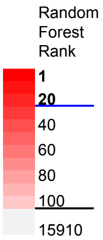

Supplementary Figure 9

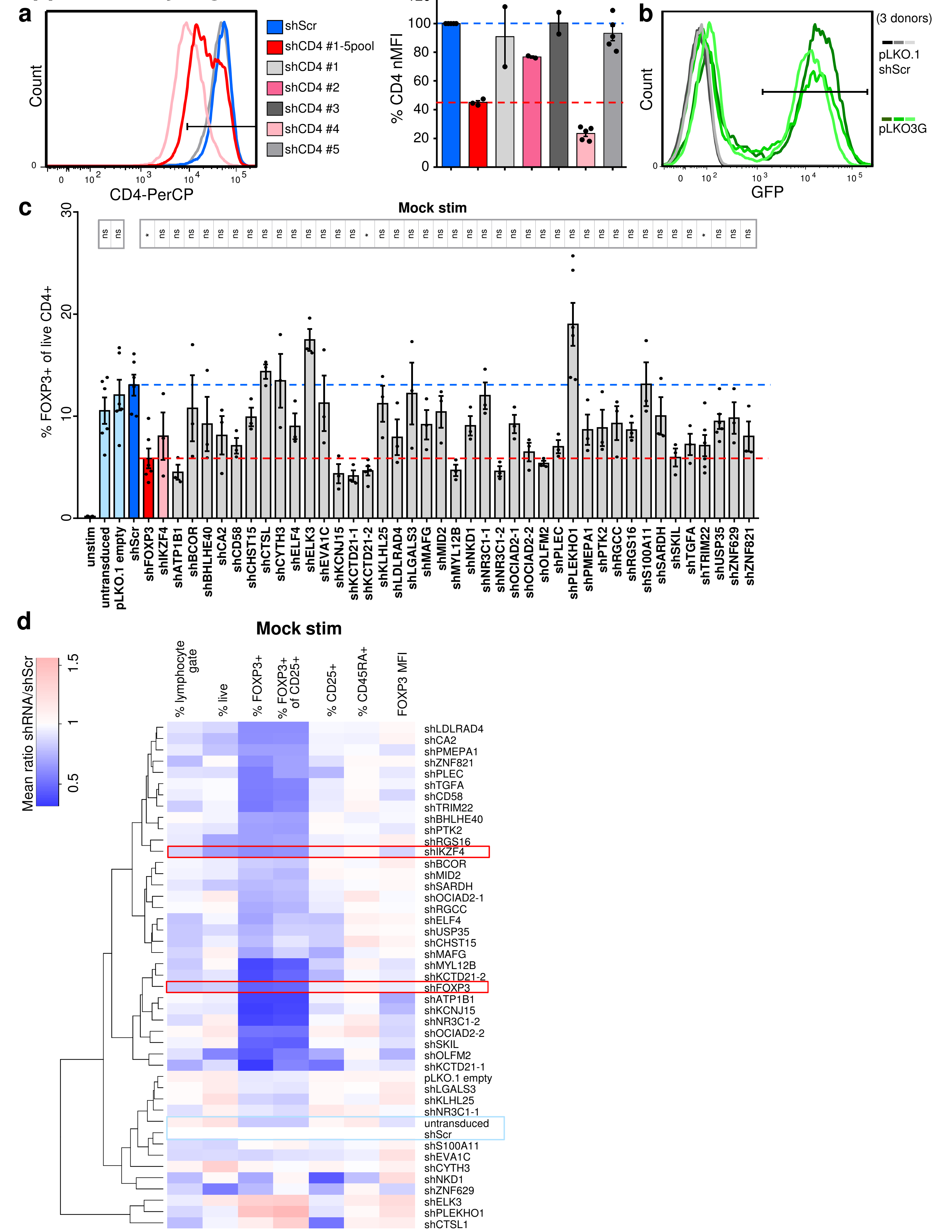

Supplement: Supplementary file 1 — Figure S1. Flow cytometry and qRT-PCR quality control of cellular samples used for molecular profiling. Figure S2. Differential gene and protein expression analysis and overlap of RNA-Seq and proteomics data. Figure S3. Exploratory analysis of RNA-Seq and proteomics data. Figure S4. Estimated number of proteins per cell based on iTreg proteomics data. Figure S5. Gene Ontology and pathway enrichment analysis of the DEG and DEP clusters. Figure S6. iTreg subnetwork reconstruction strategy. Figure S7. Features of the iTreg candidate molecules and the confirmatory independent RNA-Seq dataset. Figure S8. Linear Discriminant and Random Forest analyses confirm the potential of candidate genes to classify iTregs. Figure S9. Experimental validation of novel FOXP3+ Treg regulatory molecules. (PDF 3722 kb) [file 12915_2018_518_MOESM1_ESM.pdf]
